# Photoinduced Electron Transfer Across Phospholipid Bilayers in Anaerobic and Aerobic Atmospheres

**DOI:** 10.1002/anie.202423393

**Published:** 2025-04-26

**Authors:** Novitasari Sinambela, Richard Jacobi, Dieter Sorsche, Leticia González, Andrea Pannwitz

**Affiliations:** ^1^ Institute of Inorganic Chemistry I Ulm University Albert‐Einstein‐Allee 11 89081 Ulm Germany; ^2^ Institute of Theoretical Chemistry Faculty of Chemistry University of Vienna Währinger Straße 17 1090 Vienna Austria; ^3^ Doctoral School in Chemistry (DoSChem) University of Vienna Währinger Straße 42 1090 Vienna Austria; ^4^ Vienna Research Platform on Accelerating Photoreaction Discovery University of Vienna Währinger Straße 17 1090 Vienna Austria; ^5^ Institute for Inorganic and Analytical Chemistry (IAAC), Chemisch‐Geowissenschaftliche Fakultät Friedrich Schiller University Jena Humboldtstraße 8 07743 Jena Germany; ^6^ Center for Energy and Environmental Chemistry Jena (CEEC) Friedrich Schiller University Jena Philosophenweg 7a 07743 Jena Germany; ^7^ Helmholtz Institute for Polymers in Energy Applications Jena (HIPOLE Jena) Lessingstraße 12–14 07743 Jena Germany

**Keywords:** Artificial photosynthesis, Compartmentalization, Electron transfer, Lipid bilayer

## Abstract

In natural photosynthesis, light‐driven electron transfer across the thylakoid membrane enables efficient charge separation and the confinement of reaction spaces for generating NADPH and CO_2_ and oxidation of water. These reactions are complementary redox reactions and require different reaction conditions for optimal performance. However, current artificial photosynthesis studies only take place in the bulk and are sensitive toward oxygen and air, which limits their applicability under aerated and water‐splitting conditions. Herein, we report light‐driven electron transfer across a lipid bilayer membrane of liposome vesicles via a rigid oligoaromatic molecular wire that allows to electronically connect an oxidation and reduction reaction which are spatially separated by the membrane. The molecular wire has a simple, symmetric, easy‐to‐synthesize design based on benzothiadiazole and fluorene units and absorbs in the visible spectrum which makes it suitable for solar energy conversion. The model reactions in this study are light‐driven NADH oxidation on one side of the membrane and light‐driven reduction of an organic water‐soluble dye in the bulk phase of liposomes. Additionally, the system is active in both aerobic and anaerobic atmospheres, rendering it ideal for aerobic conditions or reactions that produce oxygen such as solar‐driven water splitting and artificial photosynthesis applications.

## Graphical abstract

Light‐driven transmembrane electron transfer across a biomimetic lipid bilayer membrane in aerobic and anaerobic atmospheres is achieved using an organic transmembrane oriented chromophore. The transmembrane molecule is designed symmetrically and easy to synthesize. This model system enables light‐driven oxidation and reduction in the different compartments of the liposome, mimicking the photosynthesis system.

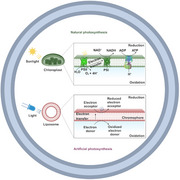

## Introduction

**Figure 1:**
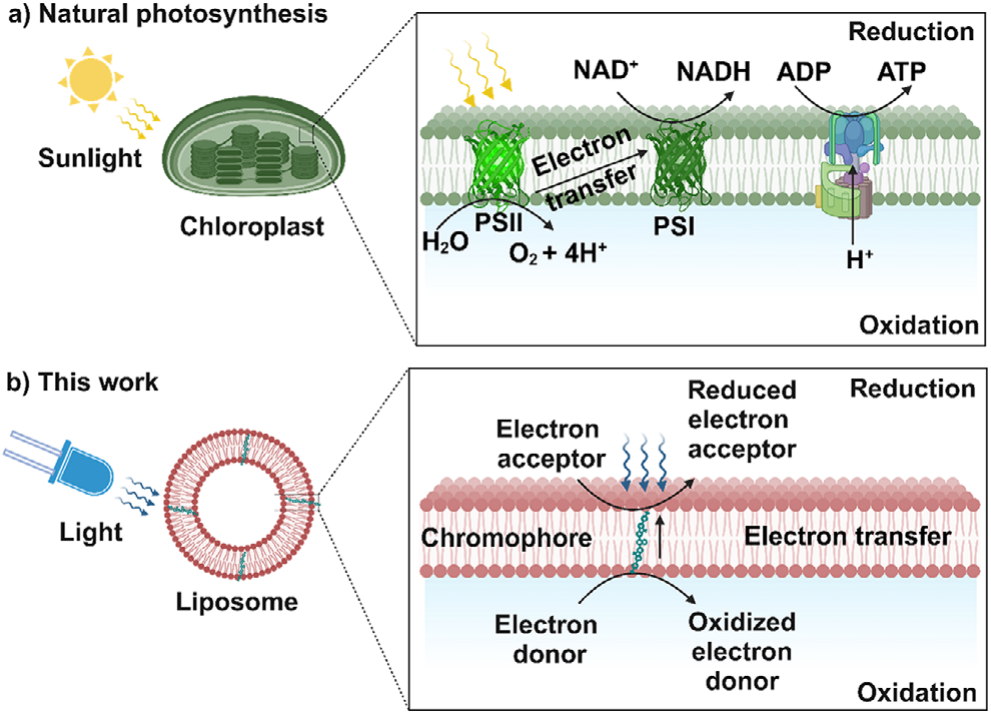
Light‐driven transmembrane in electron transfer. a) In natural photosynthesis, the light is absorbed by multiple protein supercomplexes to convert solar energy and produce NADPH and ATP (PSI = photosystem I, PSII = photosystem II). b) This work integrates a light‐active molecule for photoinduced transmembrane electron transfer in a liposome membrane to connect two spatially separated oxidation and reduction reactions. Created in https://BioRender.com.

The most relevant process of light‐energy conversion in nature is photosynthesis, which takes place within the thylakoid membrane of cyanobacteria and chloroplasts. The thylakoid membrane is a lipid bilayer that assembles multiple protein supercomplexes in a transmembrane fashion to absorb light and transfer electrons across the membrane to generate high‐energy products such as NADPH (nicotinamide adenine dinucleotide phosphate hydrogen) and carbohydrates as well as oxygen as a side product.^[^ ^1^ ^]^ The benefit of transmembrane electron transfer is that the oxidation site is physically separated from the reduction site and that such compartmentalization can fine‐tune local reaction conditions to prevent detrimental recombination.^[^ ^2^, ^3^ ^]^Additionally, the transmembrane charge transfer generates a proton gradient which fuels the ATP synthase (ATP = adenosine triphosphate, see Figure 1a).^[^ ^4^, ^5^ ^]^

Current artificial photosynthetic systems mostly investigate either an individual catalytic light‐driven reduction (e.g., H_2_ evolution,^[^ ^6^ ^]^ CO_2_ reduction^[^ ^7^ ^]^) or an individual oxidation reaction (e.g., water oxidation),^[^ ^8^, ^9^ ^]^ both of which are still relying on the use of sacrificial reagents. A useful oxidation and reduction reaction combination is challenging due to their typically different reaction conditions and reported examples also rely on sacrificial reagents.^[^ ^10^ ^]^ For instance, reduction reactions such as H_2_ evolution perform best under acidic conditions due to a favorable thermodynamic redox potential connected to proton uptake. In contrast, oxidation reactions such as water oxidation are thermodynamically optimal at basic pH as they typically involve proton release. Therefore, it is desirable to perform the reduction and oxidation reaction in different compartments while connecting the compartments electronically.

**Figure 2:**
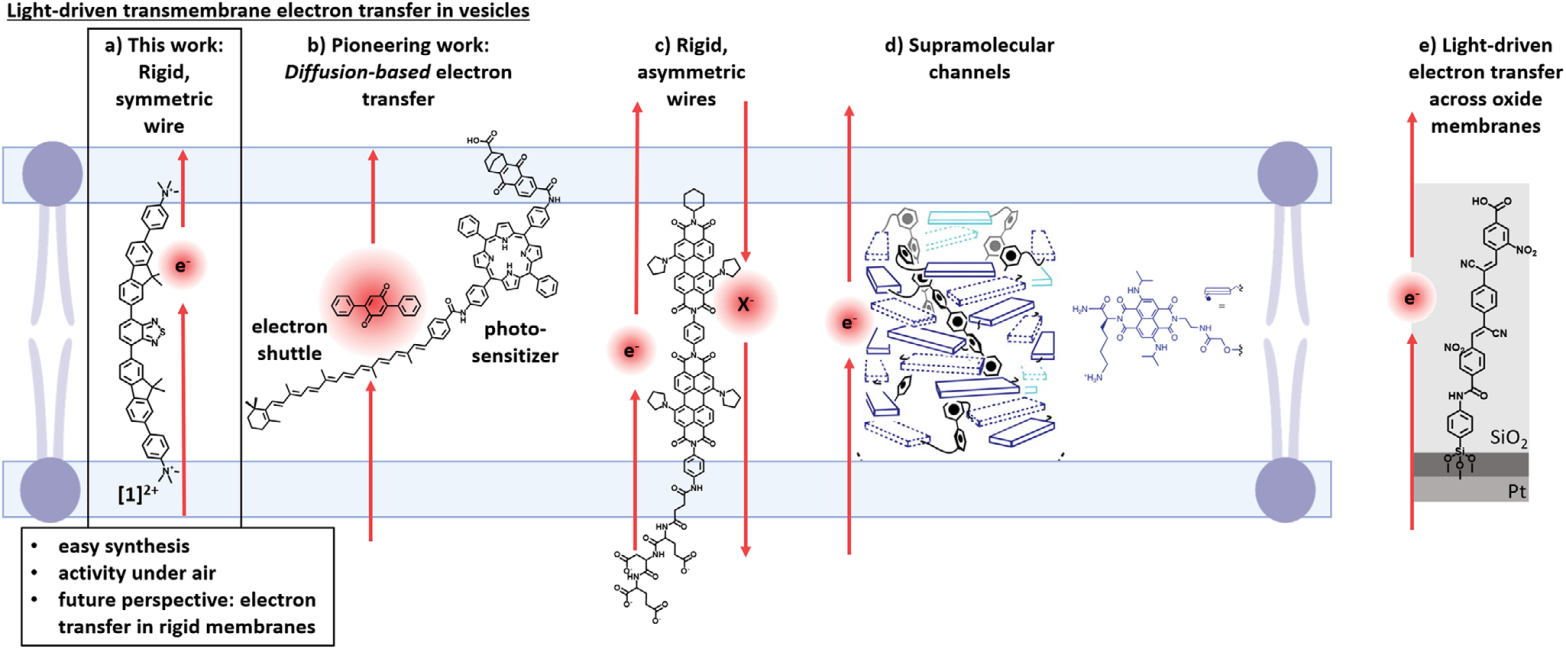
Reported systems for artificial photosynthesis: a) This work with molecular wire (**[1]^2+^**) to transfer electrons across the membrane, b) diffusion‐based light‐driven electron transfer via electron shuttle with triad harvesting complex,^[^ ^11^ ^]^ c) passive anion transport with indirect proof of the electron transfer via pH change,^[^ ^12^ ^]^ d) light‐driven electron transfer through complex supramolecular barrel like assembly, (reproduced from Ref. [13] under license number 5 911 880 595 918 from The American Association for the Advancement of Science),^[^ ^13^ ^]^ and e) molecular wire embedded in a 2‐nm‐thick SiO_2_ membrane enables electron flow from microbial catalysts to the inorganic anode.^[^ ^14^ ^]^

The system we report here uses a very simple, symmetric oligoaromatic molecular wire **[1]^2+^** that spans across the lipid bilayer membrane of artificial vesicles and effectively transfers electrons across the membrane under both inert and aerated conditions (Figures 1b and 2a). The aim is to give proof of multielectron transfer that resembles the complexity of multielectron transformation like water splitting and CO_2_ reduction. The design principle of **[1]^2+^** matches the one for membrane‐spanning molecules, having a central hydrophobic region and two terminal hydrophilic groups.^[^ ^15^, ^16^ ^]^ The distance between the terminal hydrophilic trimethylammonium groups is 3 nm and matches the thickness of the membrane to avoid destabilization of the membrane. Interaction with visible light is ensured via the benzothiadiazole at its center. Hydrophobicity is maintained by adding fluorene to its core. The symmetric design of **[1]^2+^** ensures a very easy chemical synthesis and a very defined membrane insertion. The rigid oligoaromatic design of the transmembrane molecular wire **[1]^2+^** has the potential for light‐driven electron transfer in a more rigid polymer‐based material, which will be relevant for the technological scale‐up process. As electron transfer does not rely on diffusion within the membrane, it will be possible to transfer electrons in an environment like a polymer‐based membrane.

Pioneering work on light‐driven transmembrane electron transport across vesicle bilayers was previously reported with a photosensitizer within the membrane combined with molecular electron shuttles resulting in diffusion‐based transmembrane electron transfer. A prominent example of the pioneering work is shown in Figure 2b.^[^ ^17^, ^18^, ^19^ ^]^ There are more diffusion‐based systems, which are reported for inert conditions.^[^ ^2^, ^11^, ^20^, ^21^, ^22^, ^23^, ^24^, ^25^, ^26^ ^]^ Diffusion‐independent light‐driven electron transfer has been reported for photoelectrochemical studies with *planar* lipid bilayers using the type of photosensitizer shown in Figure 2b.^[^ ^27^, ^28^ ^]^ A few elegant, but synthetically demanding rigid transmembrane molecular wires and supramolecular barrel were reported for light‐driven transmembrane electron transfer, shown in Figure 2c,d.^[^ ^12^, ^13^ ^]^ As opposed to our molecular wire **[1]^2+^** the reported asymmetric systems can insert from both sides with no preference. Apart from vesicle lipid bilayer membranes, the transmembrane design is also interesting for photoelectrochemical electron transfer and catalysis at electrode surfaces covered with an inorganic SiO_2_ membrane coating (Figure 2e).^[^ ^14^ ^]^ The function of these transmembrane electron transfer systems was to protect and connect the photo‐ and electrochemical components.^[^ ^29^, ^30^ ^]^

## Results and Discussion

### Synthesis and Characterization

**Figure 3:**
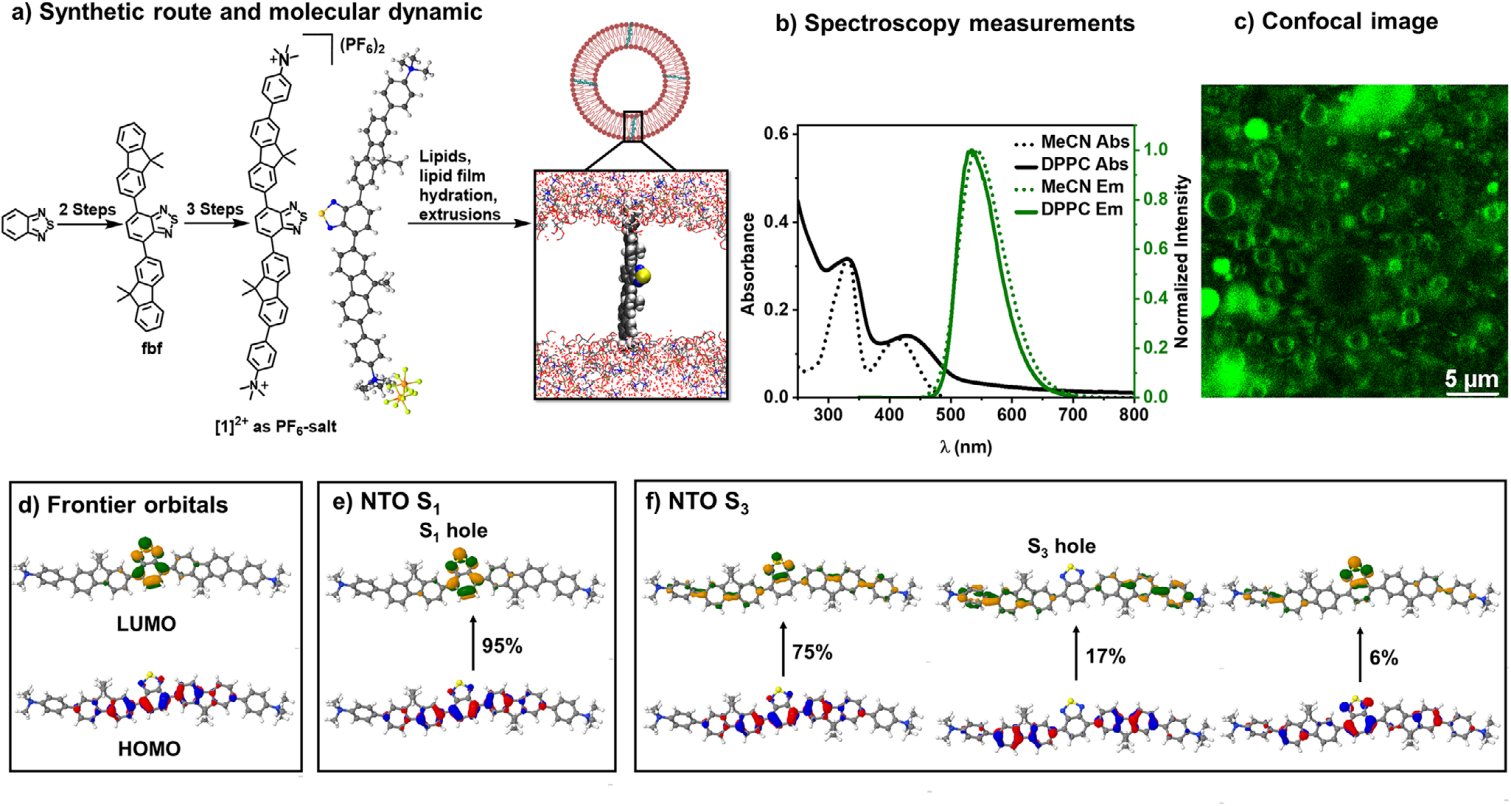
Characterization of the molecular wire **[1]^2+^**. a) Synthetic route of **[1]^2+^** as PF_6_‐salt, solid‐state structure of **[1](PF_6_)_2_** (cocrystallized solvent molecule acetonitrile omitted for clarity), and a snapshot of the molecular dynamic simulation of **[1]^2+^** in the DPPC membrane, showing how **[1]^2+^** orients within the membrane. Color code: red = oxygen, white = hydrogen, blue = nitrogen, yellow = sulfur, golden = phosphorus, and grey = carbon. The alkyl tails of the lipid bilayer are omitted for clarity. b) UV–vis absorption (Abs, in black) + emission (Em, in green) spectra of **[1]^2+^** with a ratio of DPPC:**[1]^2+^**:(14:0 PEG2000 PE) = 100:1:1; in phosphate buffer 10 mM pH 7.0 (solid line) and MeCN (acetonitrile, dot line). c) Confocal microscopy image of giant vesicles with DPPC:**[1]^2+^**:(14:0 PEG2000 PE) = 100:1:1; in phosphate buffer 10 mM, pH 7.0 after size exclusion chromatography, upon fixation in agarose hydrogel. d) Visualization of the frontier orbitals HOMO and LUMO. e), f) Natural transition orbitals (NTOs) for the S_1_ and S_3_ excitations.

The oligoaromatic light active molecule **[1]^2+^** was synthesized starting from benzothiadiazole, which was functionalized via bromination and successive two‐fold Suzuki‐cross‐coupling with dimethyl fluorene boronic acid, yielding the unpolar core **fbf** (4,7‐bis[9,9‐dimethylfluoren2‐yl]‐2,1,3‐benzothiadiazole). Subsequent bromination, Suzuki‐cross‐coupling with a boronic acid of *N*,*N*‐dimethyl aniline, followed by methylation of the nitrogen groups and ion exchange yielded the final product as PF_6_‐salt as a yellow solid in overall five steps and an overall yield of 18% (Figure 3a). The crystal structure can be found in the CCDC database with the identifier 2376492. The detailed synthesis and characterization can be found in the Supporting Information (Section S1).

For liposome preparation, DPPC (dipalmitoylphosphatidylcholin) as the main lipid, **[1]^2+^** and the PEGylated lipid 14:0 PEG2000 PE were combined in the desired molar ratio of 100 :1 :1. DPPC liposomes were chosen because they are not permeable for larger water‐soluble molecules at room temperature, which is the operating temperature during the experiments.^[^ ^17^, ^31^, ^32^ ^]^ In addition to DPPC, 1 mol% of 14:0 PEG2000 PE was added to the lipid mixture to increase stability and to prevent aggregation of the liposome via steric repulsion by the PEG chains on the liposome surface.^[^ ^22^, ^33^, ^34^ ^]^ Membrane integration of **[1]^2+^** into DPPC lipid bilayer was achieved via lipid film hydration, as described in detail in Section S2. Lipid film hydration yielded giant vesicles. When lipid film hydration was followed by extrusion, it yielded unilamellar vesicles with a typical diameter of 110 for empty liposomes and 140 nm average with encapsulated NADH according to dynamic light scattering (DLS). The size of the liposomes remained the same before and after irradiation for both cases, implying that the liposomes could retain their structure under irradiation (Section S3).

To characterize the photophysical properties and membrane integration of **[1]^2+^**, UV–vis absorption and emission spectra were recorded, and emission lifetime measurements were performed (see Figure 3b and Table 1). It was observed that **[1]^2+^** has two absorption bands in polar organic solvent (acetonitrile) at 330 and 410 nm, respectively, and one emission band at 540 nm, which decays with a lifetime of *τ* = 5.4 ± 0.5 ns, indicating a spin‐allowed fluorescence from a singlet excited state. In DPPC liposomes, the high‐energy absorption band has the same shift as in the organic solvent. By contrast, the lower‐energy band in the visible region shifts slightly bathochromically to 430 nm (Figure 3d), indicating a stabilization of the respective LUMO (lowest unoccupied molecular orbital) or destabilization of the respective HOMO (highest occupied molecular orbital), in the nonpolar membrane environment, in line with similar dyes in unpolar solvents.^[^ ^35^ ^]^ The weak tail in the red region of the liposome absorption spectrum is due to light scattering and the Tyndall effect.^[^ ^36^, ^37^ ^]^ In the emission spectra, a small hypsochromic shift toward higher energy of the emission band by 5 to 535 nm was observed, suggesting that the lowest excited state of **[1]^2+^** resides within the hydrophobic membrane core. The emission lifetime within the membrane remained similar with *τ* = 5.2 ± 0.2 ns; which is a low environmental influence and was also observed for other singlet emitters in solvents versus membrane (Supporting Information Section S4).^[^ ^34^ ^]^

**Table 1:** Spectroscopic data of **[1]^2+^**.

Solvent or lipid	*λ*_Abs_ (nm)	*λ*_em_ (nm)	*τ* (ns) Ar	*τ* (ns) air
Acetonitrile	330, 410	540	5.6 ± 0.04	5.4 ± 0.04
DPPC^a)^	330, 430	535	5.2 ± 0.1	5.2 ± 0.1

^a)^ DPPC:**[1]^2+^**:(14:0 PEG2000 PE) = 100:1:1 in phosphate buffer (10 mM, pH 7.0).

Upon incorporation into the DPPC liposomes, which are less polar compared to acetonitrile,^[^ ^38^ ^]^ the absorption band at lower energies at around 410 nm undergoes a bathochromic shift to 430 nm, while the higher energy band remains at 330 nm as in acetonitrile. To rationalize why one of the bands shifts to lower energies while the other is barely affected, we resort to time‐dependent density functional theory calculations. We used the CAM‐B3LYP^[^ ^39^ ^]^ functional and def2‐SVP^[^ ^40^, ^41^ ^]^ basis set, as implemented in Gaussian 16,^[^ ^42^ ^]^ whereby the amount of HF exchange is reduced to reproduce the experimental absorption energies.^[^ ^43^ ^]^ Further details are presented in the Supporting Information Section S5. The calculations evidence that the two absorption bands should be assigned to the S_1_ and S_3_ singlet electronic excitations, respectively, while the transition to the S_2_ is dark. The hole and electron of the excitation to the S_1_ closely resemble the shape of the HOMO and LUMO (Figure 3d). During the excitation to the S_1_ (Figure 3e), the electron density is transferred from the fluorene units to the benzothiadiazole unit. By contrast, in the S_3_ (Figure 3f), both the hole and the excited state electron are similarly delocalized over large parts of the molecule, making the S_3_ comparable in electronic properties to the ground state. Further discussion of the electronic structures in the ground and excited states can be found in the Supporting Information Section S6.

How these excited states are influenced by the environment requires elucidating the alignment of **[1]^2+^** within DPPC liposomes to judge which part of the molecule is exposed to which electrostatic environment. As the DPPC liposome is too small for confocal microscopy, we prepared giant vesicles functionalized with **[1]^2+^**, shown in Figure 3c. The fact that the membrane is selectively stained confirms that **[1]^2+^** preferably binds to the membrane. Additionally, the luminescence reveals a double‐half moon‐shaped vesicle membrane, indicating that **[1]^2+^** adopts a preferred orientation within the membrane.^[^ ^15^, ^16^, ^34^ ^]^ Furthermore, we also measure the integration of the hydrophobic chromophore core (**fbf**) in the DPPC membrane with confocal microscopy, which does not show a half‐moon shape, presumably due to a random orientation of **fbf** within the lipophilic part of the membrane. Further exploration of the alignment of **[1]^2+^** in the membrane can be found in the Supporting Information Section S7. The transmembrane alignment of **[1]^2+^** is ensured by its geometry matching with the geometry of the membrane. **[1]^2+^** has a hydrophobic core and two charged, hydrophilic N(CH_3_)_3_‐groups at a distance of 2.99 nm from one another based on crystal structure; in the structure optimized with DFT calculation the distance is 3.1 nm, and in the MD‐simulations, the distance varies between 2.8 and 3.1 nm. As the membrane thickness is typically between 3 and 5 nm, the molecule can span across lipid bilayer as the thickness of the membrane, which is also demonstrated by MD‐simulations, see a snapshot in Figure 3a and other trajectories in the Supporting Information Section S7.^[^ ^44^ ^]^ The nature of this preferred orientation is unraveled with molecular dynamics (MD) simulations (see computational setup in Section S8). In those, the photoactive molecule **[1]^2+^** is placed randomly within a simulation box together with 128 DPPC molecules solvated in water. During the simulation, the lipid bilayer self‐assembles, usually within 1–3 µs, and due to its hydrophobicity, **[1]^2+^** assembles in the newly formed membrane. The orientation of **[1]^2+^** with respect to the membrane could occur in different ways, such as in a transmembrane fashion, or parallel to the surface of the membrane as we previously found for a different chromophore in a lipid bilayer.^[^ ^34^ ^]^ However, in our simulations **[1]^2+^** aligns transmembrane, as shown in the representative MD snapshot in Figure 2a (and others in Section S8). We therefore conclude that the transmembrane configuration is the preferred alignment of **[1]^2+^** and thus responsible for the experimental signature. In detail, the ends of the molecular rod, i.e., the two trimethylammonium groups, are embedded in the opposite head group layers, which are zwitterionic and thus can be regarded as polar. The parts closer to the center of the molecule are embedded between the hydrophobic tails. As a result, the central rings of **[1]^2+^** are most exposed to the change in polarity between the polar acetonitrile and the unpolar membrane. Incidentally, this is exactly the region where both the excitations to S_1_ and S_3_ are located. As outlined above, the S_1_ exhibits a different electronic structure compared to the S_0_, and because the polar solvent will adjust to the ground state, it will stabilize the S_0_ more than an excited state of different electronic structure. When this stabilization is removed upon incorporation into the liposomes, the S_0_ will be more destabilized than the S_1_, resulting in the excitation redshift evidenced in the absorption spectra. The S_3_, by contrast, is similar to the S_0_ in electronic structure and will thus be similarly stabilized, explaining why it is much less affected by the environmental change and thus the excitation is not shifted noticeably.

### Transmembrane Electron Transfer

As a model reaction, we investigated the light‐driven oxidation of NADH within the inner aqueous compartment of the liposome vesicles and the light‐driven reduction of XTT (2,3‐bis(2‐methoxy‐4‐nitro‐5‐sulfophenyl)‐2*H*‐tetrazolium‐5‐carboxanilide sodium salt) in the outer aqueous bulk compartment. XTT is a well‐known chemical reagent in a biological assay to prove the presence of cellular reducing equivalents such as NADH, forming a strongly colored formazan dye upon reduction.^[^ ^45^ ^]^ XTT and NADH were chosen because their conversion can easily be monitored by UV–vis absorption spectroscopy,^[^ ^45^, ^46^ ^]^ as opposed to weakly absorbing Co or Fe species,^[^ ^47^, ^48^ ^]^ which would be difficult to quantify given the Tyndall scattering of the liposomes and the absorption of **[1]^2+^** itself. NADH and XTT and their oxidized and reduced products are water‐soluble and are anticipated to stay within their respective aqueous compartment in the inside or outside of the vesicel respectively, as opposed to, e.g., methyl viologen which comproportionates and diffuses across the membrane.^[^ ^49^ ^]^

**Figure 4:**
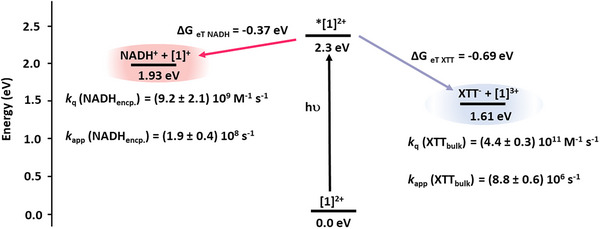
Energy profile of the redox reactions between photoexcited **[1]^2+^** and NADH or XTT, respectively, including the quenching constants *k*_q_ from the Stern–Volmer quenching studies. The apparent quenching konstants *k*_app_ quantify the quenching at the experimental conditions for transmembrane electron transfer, namely at 0.021 M encapsulated NADH and at 2 × 10^−5^ M XTT in the bulk, respectively.

**Figure 5:**
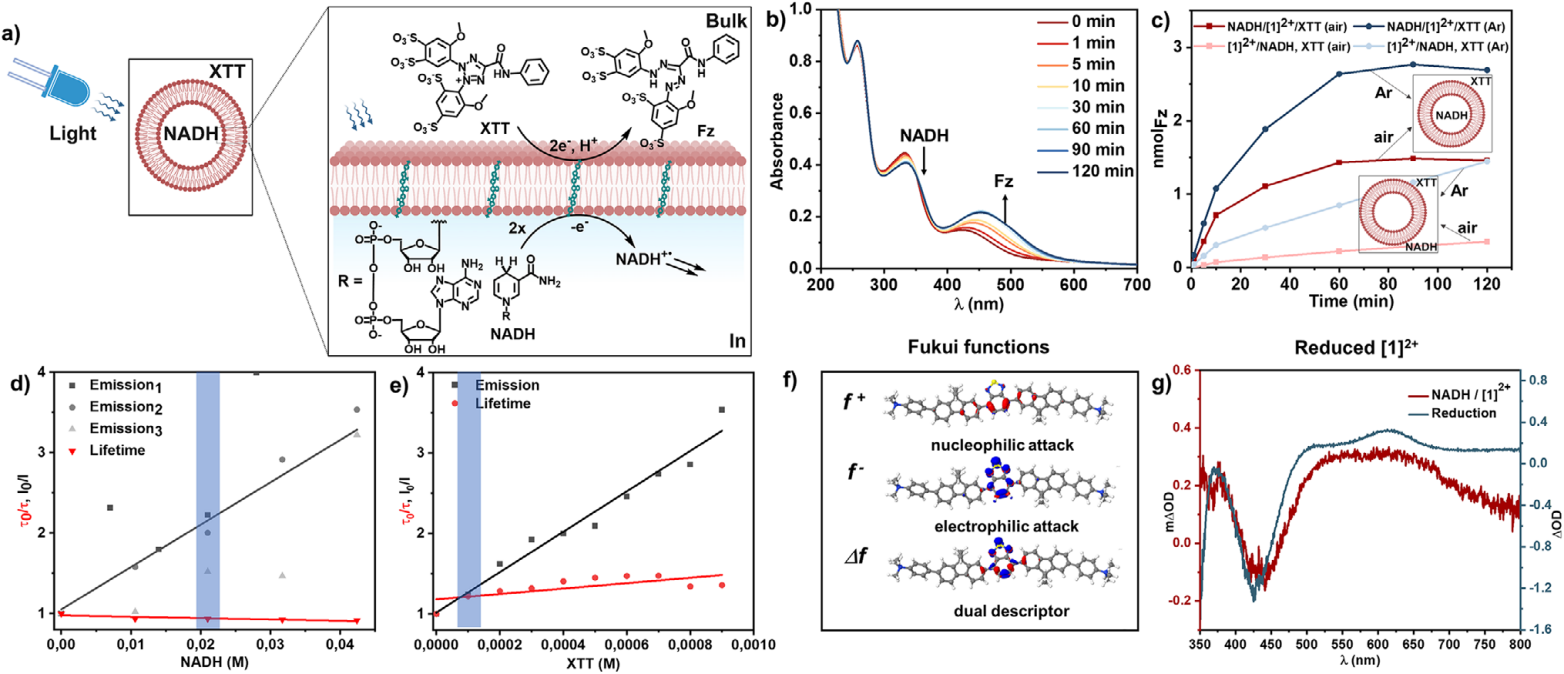
Transmembrane electron transfer. a) Schematic illustration of electron transfer across lipid bilayer with encapsulation of NADH, integration of **[1]^2+^** and XTT at the bulk solution of the liposome (anaerobic atmosphere). b) Evolution of the UV–vis absorption spectrum of DPPC:(14:0 PEG2000 PE):**[1]^2+^** = 100:1:1 with a composition of NADH/**[1]^2+^**/XTT upon irradiation with 470 nm LED light. c) Number of Fz formation under irradiation with 470 nm LED light over time for NADH/**[1]^2+^**/XTT (bold lines) and **[1]^2+^**/NADH, XTT (lighter lines) under inert and ambient atmospheres. d) Stern–Volmer quenching experiments of DPPC:(14:0 PEG2000 PE):**[1]^2+^** = 100:1:1 liposomes in phosphate buffer (10 mM, pH 7.0) with different concentrations of encapsulated NADH inside the liposome. e) Stern–Volmer quenching experiments of DPPC:(14:0 PEG2000 PE):**[1]^2+^** = 100:1:1 liposomes in phosphate buffer (10 mM, pH 7.0) with XTT as the quencher at the bulk solution of liposome. f) Fukui functions of **[1]^2+^** in the S_1_. g) Transient absorption (TA) spectra of reduce **[1]^2+^** in DPPC liposome (red line) and spectroelectrochemistry (blue line).

To enable transmembrane electron transfer across a lipid bilayer, we prepared DPPC liposomes that are impermeable to larger water‐soluble molecules^[^ ^17^, ^31^, ^32^ ^]^ and integrated 1 mol% of the amphiphilic transmembrane chromophore **[1]^2+^**, as described above. The water‐soluble electron donor NADH was added to the inner compartment of the liposome, while the water‐soluble electron acceptor XTT was placed in the bulk or on the outer part of the liposome, forming so‐called NADH/**[1]^2+^**/XTT liposomes (Figure 5a). Upon light irradiation, the absorption band at *λ*_max_ = 470 nm gradually increased, corresponding to the electron uptake of XTT to generate its reduced form formazan (Fz),^[^ ^45^ ^]^ indicating that photoinduced electron transfer had occurred (Figure 5b). Simultaneously, the NADH absorption band at 340 nm is diminished, indicating oxidation of the electron donor. However, the quantification based on the decrease of NADH absorption is not used, as the onefold oxidized NADH tends to form a dimer which also absorbs at 340 nm.^[^ ^50^, ^51^ ^]^ Leakage through the membrane was excluded through various reference experiments (Section S9), leading to the conclusion that, indeed, transmembrane electron transfer takes place. Based on the analysis of the thermodynamic properties, the excited state of **[1]^2+^** is reduced by NADH with a driving force of −0.37 eV and a driving force of −0.69 eV to oxidize by XTT. These values show that it is easier to oxidize **[1]^2+^** than to reduce it (Figure 4, the details can be found in the Supporting Information Section S10).

It was furthermore confirmed that the original NADH/**[1]^2+^**/XTT liposomes perform light‐driven transmembrane electron transfer under inert conditions with a rate constant of *k*_Ar_ = (1.76 ± 0.09) 10^−12^s^−1^ for Fz formation during the first 10 min of irradiation, and a quantum yield of *Qy* = 0.045%. The rate constant of Fz was determined by fitting the first 10 min of data with a linear function, and the *Qy* was determined by the number of Fz formed divided by the number of absorbed photons as described in Section S11. Interestingly, we found that the presence of oxygen only reduced the reaction rate and *Qy* by around factor 2 and the by a factor of 0.45 to *Qy* = 0.020%, as reported in Table 2. This observation is very promising, as oxygen normally inhibits electron transfer reactions when performed with a diffusion‐based electron shuttle.^[^ ^2^, ^22^, ^26^, ^52^ ^]^ It seems that the transmembrane molecular wire approach provides robustness, potentially enabling the investigation of transmembrane water‐splitting reactions in the future as well as reactions under aerobic conditions. Performing light‐driven electron transfer under noncompartmentalized conditions, with electron donor and acceptor in the outer bulk (**[1]^2+^**/NADH, XTT in Figure 5c and Table 2 entry 2), significantly reduced the overall electron transfer efficiency, especially under aerated conditions. This suggests that the transmembrane electron transfer is indeed more efficient than a liposome‐bulk system, and that compartmentalization might play a large role in accelerating the desired reactions, thereby minimizing the detrimental effect of oxygen. There are several reasons for such an improvement under compartmentalization conditions: (i) It enables stepwise reactions at different compartments, (ii) it allows for high local concentration of NADH in the inner part of the liposome, allowing for effective oxidation, and (iii) it prevents recombination.^[^ ^46^ ^]^

**Table 2:** Different systems of light‐driven electron transfer in inert and ambient air; reaction rates of Fz formation at initial *t* = 10 min and initial quantum yield (*Qy*).

Entry	Conditions	*k*_air_ Fz air (10^−12^ s^−1^)	*k*_Ar_ Fz Ar (10^−12^ s^−1^)	*Qy* air (%)	*Qy* Ar (%)
1	NADH/**[1]^2+^**/XTT	1.18 ± 0.01	1.76 ± 0.09	0.020	0.044
2	**[1]^2+^**/NADH, XTT	0.13 ± 0.01	0.50 ± 0.02	0.001	0.012
3	NADH/**[1]^2+^**, 1% gramicidin A/XTT	1.50 ± 0.02	1.95 ± 0.04	0.030	0.053
4	NADH/**[1]^2+^**, 4.2% 18‐crown‐6/XTT	0.60 ± 0.01	0.63 ± 0.02	0.011	0.014

Reference measurements for the stability of **[1]^2+^** during 2 h of irradiation, oxidation of encapsulated NADH, bulk oxidation of NADH, bulk reduction of XTT with and without **[1]^2+^**, and bulk oxidation and reduction of XTT and NADH, as well as measurements of NADH/**[1]^2+^**/XTT and the opposite combination XTT/**[1]^2+^**/NADH, the core of dye **fbf** with a combination of NADH/**fbf**/XTT and NADH/**[1]^2+^**/XTT under aerobic and anaerobic atmosphere at an elevated temperature close to the transition temperature and experiments with different loading of **[1]^2+^** can be found in Section S11. The reverse combination, XTT/**[1]^2+^**/NADH, showed that the photoinduced transmembrane electron transfer across the membrane can also occur in the reverse configuration. The respective data are shown in Supporting Information Figures S33 and S39, showing comparable Fz formation compared to the normal system (2.53 nmol (XTT/**[1]^2+^**/NADH) vs. 2.68 nmol (NADH/**[1]^2+^**/XTT)), which highlights the adaptability of the system. An inherent limitation of our transmembrane electron transfer design is that a transmembrane potential can build up, which ultimately stops the transmembrane electron transfer. To balance the buildup of charges across the membrane, we tested two transmembrane ion transporters, gramicidin A and 18‐crown‐6 ether (also see Table 2, entries 3,4). Gramicidin A is an ion‐channel‐forming peptide, selective for small ions, such as protons.^[^ ^53^ ^]^ Under ambient aerated conditions, the initial quantum yield of gramicidin A was reduced and performed better in an inert atmosphere compared to the same system without gramicidin A. It was observed that gramicidin A played a role as an ion balancer, leading to an increase in initial quantum yield and an improvement in reaction rate. Gramicidin A accelerated the electron transfer rate constant by 11% under Ar and 27% under air. The quantum yield in the first 10 min is increased by 20% under Ar and by 50% under air. Another transmembrane ion transfer reagent 18‐crown‐6 ether was incorporated into the membrane. This transporter is selective only to K^+^ cations.^[^ ^54^ ^]^ Adding 18‐crown‐6 to the phosphate buffer in the experiment enables the transport of K^+^ cations to compensate for the charge imbalance in both compartments. It was observed that the addition of 18‐crown‐6 did not result in a better rate or quantum yield of transmembrane transfer compared to the addition of gramicidin A.

Upon photoexcitation of **[1]^2+^**, the photoredox reactions take place from the excited state of **[1]^2+^**.

To explore the photoinduced electron transfer in DPPC liposomes, the prepared samples of integrated **[1]^2+^** in DPPC, **[1]^2+^** in DPPC with encapsulated NADH (NADH/**[1]^2+^**), and **[1]^2+^** in DPPC with XTT placed at the bulk (**[1]^2+^**/XTT), and the combination of encapsulated NADH and XTT in bulk (NADH/**[1]^2+^**/XTT) respectively, were excited at 420 nm with ∼10 ns laser pulses (all the temporal spectra in different timescales and temporal kinetics can be found in the Supporting Information Section S12). According to the transient absorption (TA) spectra, both the reduction and the oxidation of **[1]^2+^** are happening: The spectral features of reduced **[1]^2+^** can be found in the transient absorption spectrum of NADH/**[1]^2+^** as well as in the spectroelectrochemical UV–vis spectrum of reduced **[1]^2+^** as a broad absorption band between 500 and 700 nm (see Figure 5g). Under oxidative conditions and upon photoirradiation, the formazan photoproduct Fz accumulates and obstructs the transient absorption measurements as it creates a bleach at the Fz absorption wavelength at 500 nm. The same artifact was also observed in the sample composition of NADH/**[1]^2+^**/XTT, making it challenging to evaluate the photoexcited species in this composition (see Supporting Information Figure S40).

To elucidate the electron transfer dynamics of **[1]^2+^**, we conducted time‐resolved and steady‐state emission spectroscopy. In an emission quenching assay, known as the Stern–Volmer plot,^[^ ^55^ ^]^ it was observed that the photoexcited state of **[1]^2+^** was quenched at varying concentrations of NADH and XTT (Table 3, spectra and kinetic traces can be found in Section S13). NADH, acting as a reductive quencher, showed a Stern–Volmer constant with different values for quenching of the emission spectrum's intensity and lifetime, with *K*_SV_(*I*_0_/*I*) = 47.8 ± 11 M^−1^ and *K*_SV_ (*τ*_0_/*τ*) = −2.2 ± 0.8 M^−1^, respectively. As the lifetime‐based *K*_SV_ (*τ*_0_/*τ*) is close to zero, a static quenching process is active, indicating the association of cationic membrane‐bound photosensitizer and anionic NADH through electrostatic attraction at the ground state (Figure 5d). The same behavior was also observed in the bulk experiment with NADH as a quencher with *K*_SV_(*I*_0_/*I*) = 7.3 ± 1.0 M^−1^and *K*_SV_ (*τ*_0_/*τ*) = 0.6 ± 0.4 M^−1^, the lifetime based on *K*_SV_ (*τ*_0_/*τ*) is close to zero (the Stern–Volmer plot this experiment can be found in Supporting Information Section S13). On the other hand, XTT, acting as an oxidative quencher, showed a mixture of dynamic and static quenching processes, with the intensity‐based and lifetime‐based Stern–Volmer constants being positive, with *K*_SV_(*I*_0_/*I*) = (2.2 ± 0.2) 10^3^ M^−1^and *K*_SV_ (*τ*_0_/*τ*) = (3.0 ± 1.1) 10^2^ M^−1^, respectively. These constants indicate excited state electron transfer upon the diffusional encounter between the liposome‐bound **[1]^2+^** and XTT and also through the association of the anionic XTT with membrane‐bound photosensitizer at the ground state (Figure 5e).^[^ ^7^, ^33^, ^34^ ^]^ The quenching constants *k*_q_ were calculated from the Stern–Volmer constants *K*_SV_ via the relationship *K*_SV_ = *k*_q_ · τ_0_.^[^ ^55^ ^]^ The intensity‐based values yield very hiqh quenching constants on the order of 10^9^–10^11^ M^−1^ s^−1^, probably due to the ground‐state association. Interestingly, the quenching constant *k*_q_ by XTT in the bulk is more efficient than by encapsulated NADH, in line with the 0.32 eV higher driving force for oxidative versus reductive quenching (Figure 4). However, under the experimental conditions of transmembrane electron transfer with 0.021 M encapsulated NADH and 2 × 10^−5^ M XTT in the bulk, the apparent quenching constant *k*_app_ is much lower for quenching by XTT compared to quenching by encapsulated NADH *k*_app_ (NADH_encp._) = (1.9 ± 0.4) 10^8^ s^−1^ and *k*_app_ (XTT_bulk_) = (8.8 ± 0.6) 10^6^ s^−1^.

**Table 3:** Stern–Volmer quenching constants of **[1]^2+^** in DPPC membrane with NADH and XTT as quenchers.

Quencher	*K*_SV_ from *I*_0_/*I* (M^−1^)	*K*_SV_ from *τ*_0_/*τ* (M^−1^)	*k*_q_ from *I*_0_/*I* (M^−1^s^−1^)	*k*_q_ from *τ*_0_/*τ* (M^−1^s^−1^)	Quenching Mechanism
NADH_encp._	47.8 ± 11	−2.2 ± 0.8	(9.2 ± 2.1) 10^9^	(−4.2 ± 1.6) 10^8^	Static
NADH_bulk_	7.3 ± 1.0	0.6 ± 0.4	(1.4 ± 0.2) 10^9^	(1.1 ± 8.0) 10^8^	Static
XTT_bulk_	(2.2 ± 0.2) 10^3^	(3.0 ± 1.1) 10^2^	(4.4 ± 0.3) 10^11^	(5.8 ± 2.1) 10^10^	Dynamic/static

To elucidate further on what causes these different quenching constants and which molecule parts of **[1]^2+^** are involved in the charge transfer, we computed Fukui functions in the S_1_ excited state (Section S5). The Fukui descriptor is a spatially resolved measure of the interaction between a molecule and either a nucleophile or an electrophile. It thus describes the change in the electronic wave function upon oxidation or reduction. Upon photoexcitation of **[1]^2+^**, the photo redox reactions take place from the excited state of **[1]^2+^**. The Fukui function for a nucleophilic attack is defined as the difference between the electron density of the molecule in the initial state with N electrons and the electron density after reduction with an additional electron: *f* ^+^ = ρ_*N* + 1_ − ρ_*N*_. Analogously, the Fukui function for an electrophilic attack is the difference in density before and after oxidation: *f* ^−^ = ρ_*N*_ − ρ_*N* − 1_. The Fukui functions for **[1]^2+^** are shown in Figure 5f. In blue, *f* ^−^ shows the areas where **[1]^2+^** would donate an electron, and thus be oxidized (by XTT), while *f* ^+^ shows those where it would accept an electron to be reduced (by NADH). According to these Fukui functions, **[1]^2+^** would be oxidized in the central moiety with the electron mostly stemming from the sulfur atom, and it would be reduced mostly into the six‐membered ring of the central moiety. To weigh the oxidative and reductive properties of **[1]^2+^**, we also compute the so‐called dual descriptor, which is the difference between the Fukui functions of an oxidation and a reduction: Δ*f* = *f* ^+^ − *f* ^−^, also shown in Figure 5f. It prominently exhibits the character of *f* ^−^, which means that the electron donating properties of the central moiety overweigh the electron accepting character. In other words, it is easier to oxidize **[1]^2+^** than to reduce it which is in line with the experimentally determined redox potentials (*E*_ox_ = 1.0 V vs. Fc/Fc^+^ and *E*_red_ = −1.7 V vs. Fc/Fc^+^ in acetonitrile, see Table S2 and Figure S20). It is also in line with the Stern–Volmer constants being so much larger for quenching by XTT compared to quenchning by NADH, which is also in line with the respective driving force for (see Figure 4).

**Figure 6:**
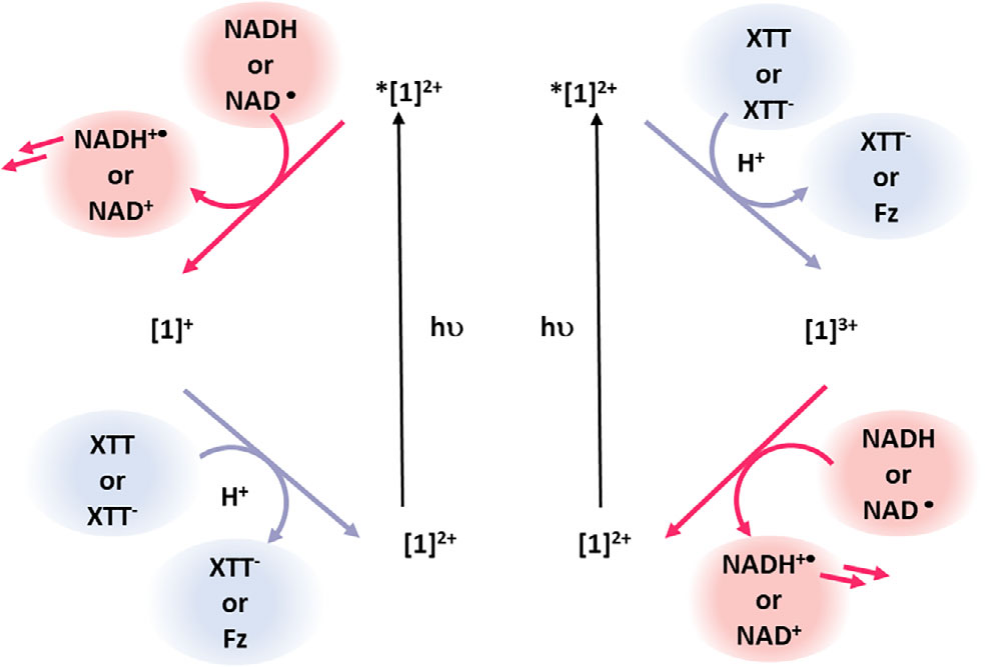
Proposed mechanism of electron transfer under inert conditions. Potential comproportionation of **[1]^3+^** with **[1]^+^** to **[1]^2+^** is omitted for clarity.

We propose the following overall mechanism, also schematized in Figure 6: Upon photoexcitation to generate *[1]^2^⁺ excited state charge transfer takes place with NADH as well with XTT in individual subsets of *[1]^2^⁺, generating the oxidized and reduced **[1]^2^⁺** within the membrane. Although the excited state electron transfer to XTT might be dominant, we cannot rule out excited sate electron transfer from NADH, as both processes are very fast based on the Stern–Volmer data and the transient absorption spectrum shown in Figure 5. The oxidized and reduced versions of **[1]^2^⁺** might then comproportionate yielding two equivalents of **[1]^2+^**, or react further with the substrates in the aqueous phases. **[1]^3+^** can oxidize a second equivalent of NADH, and **[1]^+^** can reduce the photogenerated XTT^−^ a second time. The generated NADH^+•^ is known to quickly loose its proton and dimerize.^[^ ^50^, ^51^ ^]^ Unreacted NAD^•^ can undergo a second electron donation to either **[1]^3+^** or *[1]^2^⁺. In the presence of oxygen, photo‐oxidation or photoreduction may be mediated by photogenerated singlet oxygen (Supporting Information, Figure S55).^[^ ^45^, ^56^ ^]^

### Conclusions and Outlook

In summary, we report a very simple and novel design of a photoactive molecular wire for transmembrane electron transfer using visible light under inert and aerated conditions. We demonstrate, with confocal spectroscopy and molecular dynamic simulations, that **[1]^2+^** integrates with transmembrane orientation in the lipid bilayer. Light‐driven electron transfer dynamics across the membrane were verified with two model redox reactions, and the excited state, photoreduced, and photooxidized products were characterized, along with the static and dynamic/static character of the initial photoinduced electron transfer. Overall, the liposomal system allowed us to electronically connect two complementary redox reactions through visible light‐driven electron transfer. This process is crucial for solar energy conversion and the development of more advanced systems with robust and sturdy membranes. Additionally, it is important for the design of artificial cells and cellular compartments. Although the challenge of resupplying the inner aqueous compartment with substrate still needs to be solved, the here presented findings on nanoreactors and transmembrane electron transfer are valuable for designing chemical synthetic methodologies involving compartmentalization. To create a larger photoreactor, the same approach can be applied to polymer‐based vesicles or larger planar membranes. The biorelated nature of the lipid bilayer membrane also offers opportunities for incorporating enzymatic catalysis, as the lipid membrane's complex units can be organized to carry out specific functions.

## Methods

### The General Procedure of Liposome Preparation

To a 5 mL round bottom flask was added 1 mL of DPPC (5 mM in CHCl_3_), 1 mL of 14:0 PEG2000 PE (0.05 mM in CHCl_3_), and 1 mL of **[1]^2+^** (PF_6_)_2_ (0.05 mM in acetonitrile). The organic solvents were evaporated under vacuum leading to deposition of lipids on the flask wall. The film was dried under high vacuum for at least 1 h and hydrated with phosphate buffer pH of 7.0 and added 15 mg of NADH. The dispersed lipid film was repeatedly freeze‐thawing, using liquid N_2_ and a water bath at 52 °C, forming the giant vesicles. To obtain liposomes of uniform size, the dispersion was extruded at 52 °C through 200 nm cellulose membrane filters 11 times with an Avanti Polar Lipids mini‐extruder. The liposome mixture was subjected to a Sephadex G‐25 size exclusion chromatography (SEC) column (6 cm length, 2 cm diameter) using phosphate buffer pH 7.0 as eluent; the liposome loaded with NADH was collected as the first band.

### The General Procedure of Photoirradiation

The typical reactions were carried out within quartz cuvettes where 300 µL of liposome solution, 10 µL of 1 mM XTT, and 190 µL phosphate buffer pH 7.0 were added. The mixtures were illuminated within a custom‐made reactor equipped with four ventilators to exclude heating of the samples and an LED‐stick (Nichia Corporation; NSPB500AS) (*λ* = 470 ± 10 nm, 15.7 mW measured with a Hioki 3664 optical power meter set to 470 nm).^[^ ^57^ ^]^ The samples were monitored via UV–vis spectroscopy after 0, 1, 5, 10, 30, 60, 90, and 120 min.

## Conflict of Interests

The authors declare no conflict of interest.

## Supporting information



Supporting‐Information

Supporting‐Information

Supporting‐Information

## Data Availability

The data that support the findings of this study are available in the Supporting Information of this article.
