# Supplementary material for: Photoinduced Electron Transfer Across Phospholipid Bilayers in Anaerobic and Aerobic Atmospheres
Source: Angew Chem Int Ed Engl. 2025 Apr 26;64(22):e202423393. doi: 10.1002/anie.202423393 (PMC12105711; doi:10.1002/anie.202423393)
Supplement: Supplementary file 1 — Supporting‐Information [file ANIE-64-e202423393-s002.pdf]

Supporting Information

Photoinduced Electron Transfer across Phospholipid Bilayers in Anaerobic and Aerobic Atmospheres

Authors:

Novitasari Sinambela+,[a] Richard Jacobi+,[b,c] Dieter Sorsche,[a] Leticia González*,[b,d] Andrea Pannwitz* [a,e,f,g]

[a] Institute of Inorganic Chemistry I, Ulm University, Albert-Einstein-Allee 11, 89081 Ulm, Germany

[b] Institute of Theoretical Chemistry, Faculty of Chemistry, University of Vienna, Währinger Straße 17, 1090 Vienna, Austria

[c] Doctoral School in Chemistry (DoSChem), University of Vienna, Währinger Straße 42, 1090 Vienna, Austria

[d] Vienna Research Platform on Accelerating Photoreaction Discovery, University of Vienna, Vienna, Austria, Währinger Straße 17, 1090 Vienna

[e] Institute for Inorganic and Analytical Chemistry (IAAC), Chemisch-Geowissenschaftliche Fakultät, Friedrich Schiller University Jena, Humboldtstraße 8, 07743 Jena

[f] Center for Energy and Environmental Chemistry Jena (CEEC), Friedrich Schiller University Jena, Philisophenweg 7a, 07743 Jena

[g] Helmholtz Institute for Polymers in Energy Applications Jena (HIPOLE Jena), Lessingstraße 12–14, 07743 Jena, Germany

+ These authors contributed equally

Table of Contents

S1. Experimental details 3

S2. Liposome preparation 12

S3. The typical hydrodynamic diameter of DPPC liposome containing $[1]^{2+}$ 13

S4. Lifetime of $[1]^{2+}$ in different environments. 14

S5. Computational details for the computation of electronic excited states and Fukui functions 14

S6. Characterization of the excited states 17

S7. Confocal microscopy 19

S8. Computational details on the membrane assembly simulations 20

S9. Calceine-luminescence leakage test 22

S10. Electrochemical and Spectroelectrochemical characterization 24

S11. Photoirradiation 29

S12. Nanosecond transient absorption spectra 36

S13. Stern-Volmer quenching experiment 41

S14. Proposed mechanism in the presence of oxygen, photooxidation or photoreduction 45

S15. Coordinates of $[1]^{2+}$ 46

References 47

S1. Experimental details

All reagents were commercially available and all inert synthesis reactions were carried out using standard Schlenk-techniques and argon gas. A Bruker DRX 400 spectrometer was used to record nuclear magnetic resonance (NMR) at 298 K. Mestre Nova software was used for the evaluation of the spectra. The mass spectra of the compounds were obtained using matrix-assisted laser desorption ionization (MALDI) Bruker Reflex III mass spectrometer. Trans-2-[3-(4-tert-Butylphenyl)-2-methyl-2-pro-penylidene] malononitrile was used as a matrix. Elemental analysis was performed by Mikroanalytisches Laboratorium Kolbe in Oberhausen, Germany. The elemental content of the molecules was reported as the element's mass fraction percentage. Absorptions were recorded on a spectrometer from JASCO V760. Starna fluorescence quartz cuvettes with a path length of 1 cm and screw caps with an air-tight silicon seal were used. Luminescence was recorded on a FP-8500 Spectrofluorometer. The size distribution of the hydrodynamic diameter (Z_{Avg}) was measured at 20 °C with a Zetasizer Pro from Malvern operating at 633 nm with a scattering angle of 173°. Confocal microscopy images were acquired on a Leica TCS SP8 confocal microscope equipped with a 488 nm laser as excitation source and detected with range of 500-600 nm. Sample wells (μ-Slide 8 Well ibiTreat) were used to characterize giant vesicles. Confocal microscopy images were processed with the LasX software. DC cyclic voltammetry (CV) experiments were performed on a Pine Research Wavedriver 200 electrochemical workstation equipped with a standard three-electrode arrangement: working electrode (WE): glassy carbon electrode (d = 3.0 mm), quasi reference electrode (RE): Ag wire (in a glass frit containing electrolyte solution), counter electrode (CE): Pt wire. All potentials are quoted relative to the ferrocene/ferrocenium internal standard. All experiments were performed in dry dimethylformamide (DMF) and acetonitrile (MeCN), using nBu_4NPF_6 (0.1 M) as supporting electrolyte. The solutions were purged with argon for at least 15 minutes to remove O_2 and kept under a slight positive argon pressure while performing the experiments. Spectroelectrochemical measurements were performed using a Pine honeycomb screen-printed platinum electrode as WE and CE and an Ag wire (in a glass frit containing electrolyte solution) as pseudo-RE. Nanosecond transient absorption experiments were performed on an LP980-K spectrometer from Edinburgh Instruments equipped with an iCCD detector from Andor (DH320T-25F-03-812), a monochromator (STGM325-MA) and a photomultiplier (PMT-LP R928P). The excitation source was an Nd: YAG/YVO4 laser from Ekspla (NT342B-10-AW) equipped with a tunable OPO (410-2600 nm). The sample chamber was tempered to 20 °C using a built-in thermostat. For all spectral measurements, 20 pulses were averaged and the signal was integrated over a 100 ns timespan.

Synthesis route

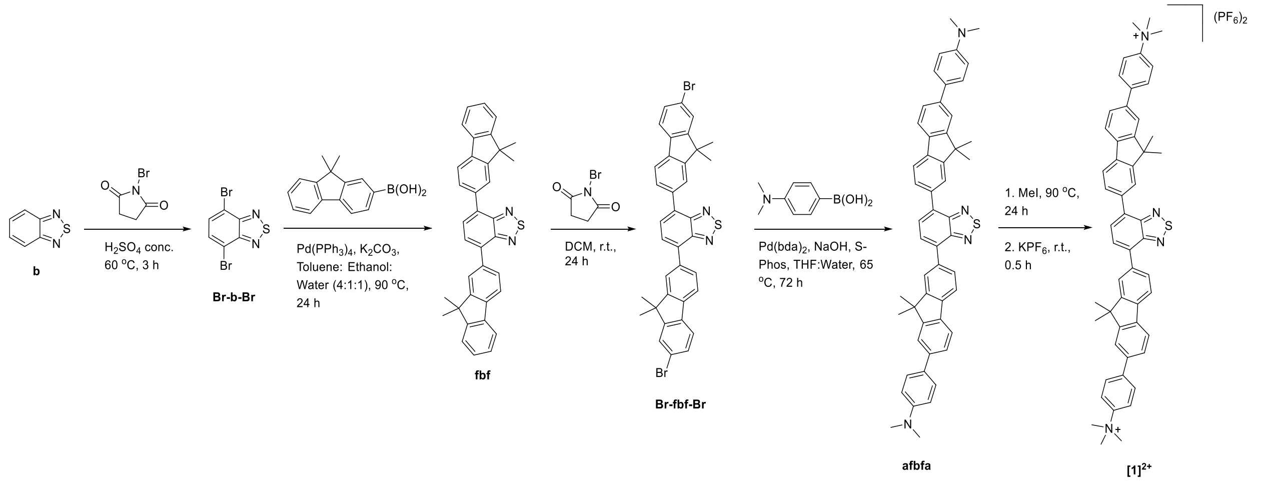

Figure. S1. Synthesis route of molecule **[1]**$^{2+}$.

4,7-Dibromo-2,1,3-benzothiadiazole (Br-b-Br)

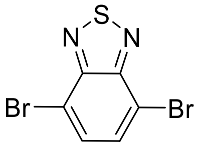

Br-b-Br

In a water bath 2,1,3-benzothiadiazole (1.0 g, 7.34 mmol, 1.00 equivalent (eq.)) and N-bromosuccinimide (3.3 g, 18.4 mmol, 2.52 eq.) were added in a round-bottom flask. Concentrated H_2SO_4 (97%, 10 mL) was added dropwise and the reaction mixture was stirred for 3 h at 60 °C. The reaction cooled to room temperature and placed in an ice bath. To the reaction mixture a 100 ml of distilled water was added dropwise. The product was extracted with 3 x 30 mL of toluene and the combined organic layers were dried with Na_2SO_4 and evaporated under reduced pressure. The reaction gave compound **Br-b-Br** as white-yellow powder (2.10 g, 7.14 mmol) in 97% yield.

^{1}H-NMR (400 MHz, $CDCl_3$): δ = 7.72 (s, 2 H) ppm.

^{13}C-NMR (200 MHz, $CDCl_3$): δ = 152.98, 132.36, 113.93 ppm.

4,7-bis[9,9-dimethylfluoren2-yl]-2,1,3-benzothiadiazole (fbf)

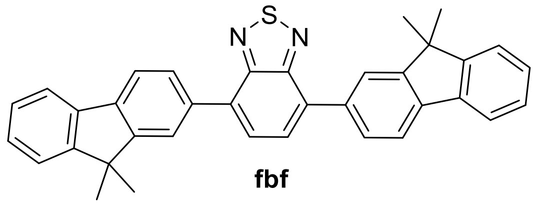

fbf

To a mixture of 4,7-dibromo-2,1,3-benzothiadiazole (0.5 g, 1.70 mmol, 1.00 eq.), 9,9-dimethyl-9H-fluoren-2-yl-2-boronic acid (0.89 g, 3.74 mmol, 2.20 eq.) and K_2CO_3 (1.88 g, 13.6 mmol, 8.00 eq.) in a pressure flask, a degassed solution mixture of 40 mL toluene, 10 mL ethanol and 10 mL water was added. A catalyst $Pd(PPh_3)_4$ (0.17 g, 0.17 mmol, 0.1 eq.) was added under counter flow of argon and the reaction mixture was stirred at 90 °C for 24 h, after the completion of the reaction yellow crystals were forming. The crystals were filtered and washed with water, ethanol, and Et_2O. The crystals were dried under vacuum. Yielding **fbf** as yellow-green powder (0.59 g, 1.13 mmol) in 66% yield.

UV-vis ($CHCl_3$): λ_{max} = 259 nm, 319 nm, and 415 nm.

Luminescence ($CHCl_3$) λ_{ex} at 415 nm: λ_{max} = 550 nm.

^{1}H-NMR (400 MHz, $CDCl_3$): δ = 8.04 - 8.01 (m, 4 H), 7.91 – 7.89 (d, 4 H), 7.82 – 7.80 (m, 2 H), 7.50 – 7.48 (m, 2 H), 7.41 – 7.34 (m, 4 H), 1.60 (s, 12 H) ppm.

^{13}C-NMR (200 MHz, $CDCl_3$): δ = 154.30, 154.11, 154.00, 139.52, 138.82, 136.47, 133.57, 128.42, 128.03, 127.53, 127.10, 123.62, 122.68, 120.30, 120.10, 47.08, 27.25 ppm.

4,7-bis[7-dibromo-9,9-dimethylfluoren-2yl]-2,1,3-benzothiadiazole (Br-fbf-Br)

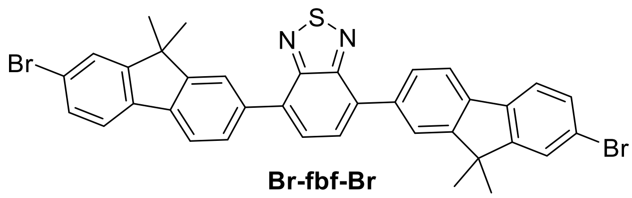

Br-fbf-Br

In a pressure flask a solution of compound **fbf** (0.35 g, 0.67 mmol, 1.00 eq.) in dichloromethane was degassed with argon and N-bromosuccinimide (0.27 g, 1.55 mmol, 2.26 eq.) was added. The mixture was stirred for 24 h at room temperature. After 24 hours, 10 mL of 6 M HCl was added and the mixture was stirred for an additional 2 hours, resulting in the formation of a yellow solid precipitate. The mixture was passed through a filter to separate the solid from the liquid. Then, the solid was rinsed with water and diethyl ether (Et_2O). The yellow powder of **Br-fbf-Br** (0.23 g, 0.34 mmol) was obtained by drying the solid under vacuum with a 50% yield.

^{1}H-NMR (400 MHz, $CDCl_3$): δ = 8.02 – 7.99 (m, 4 H), 7.88 – 7.86 (m, 4H), 7.67 – 7.65 (m, 2 H), 7.61(d, *J* = 1.7 Hz, 2 H), 7.53 (dd, *J* = 1.8 Hz, 2 H), 1.65 (s, 12 H) ppm.

4,7-bis[7-p-N,N-dimethyl-aniline-9,9-dimethylfluoren-2-yl]-2,1,3-benzothiadiazole (afbfa)

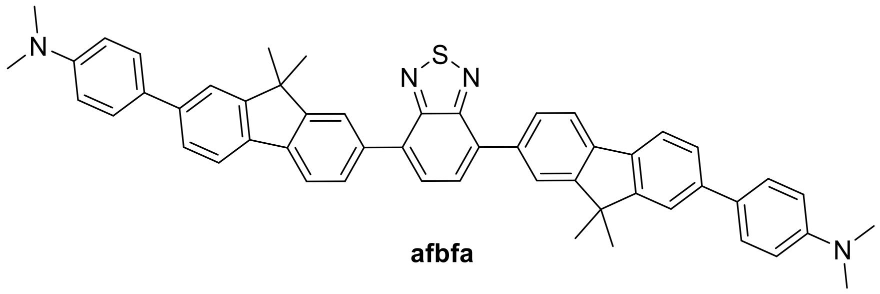

afbfa

Br-fbf-Br (0.3 g, 0.44 mmol, 1.00 eq.), N, N-dimethylaniline boronic acid (0.17 g, 1.02 mmol, 2.31 eq.), NaOH (0.74 g, 18 mmol, 40.9 eq.), and S-Phos (dicyclohexyl(2,6-dimethoxy[1,1-biphenyl]-2-yl) phosphane) (0.04 g, 0.1 mmol, 0.22 eq.) were suspended in a deoxygenated mixture of tetrahydrofuran (130 mL) and water (5 mL). The suspension was degassed with argon for 30 minutes before addition of $Pd_2(dba)_3$ (0.042 g, 0.04 mmol, 0.1 eq.). The mixture was heated and stirred at 65 °C for 3 days. After cooling down to room temperature, some orange precipitate was formed. The precipitate was then filtered off and washed with toluene, water and Et_2O. The powder obtained was dissolved in chloroform and recrystallized with methanol. As a result, orange powder was produced and it gave **afbfa** (0.25 g, 0.33 mmol) in a yield of 74%.

^{1}H-NMR (400 MHz, $CDCl_3$): δ 8.06 – 8.01 (m, 4H), 7.90 (t, *J* = 3.9 Hz, 4H), 7.82 (d, *J* = 7.9 Hz, 2H), 7.61 (m, 8H), 6.86 (d, 2H), 3.03 (s, 12H), 1.64 (s, 12H).

4,7-tris[7-p-N, N, N-trimethyl-aniline-9,9-dimethylfluoren-2-yl]-2,1,3-benzothiadiazole dihexafluorophosphate $[1]^{2+}(PF_6)_2$

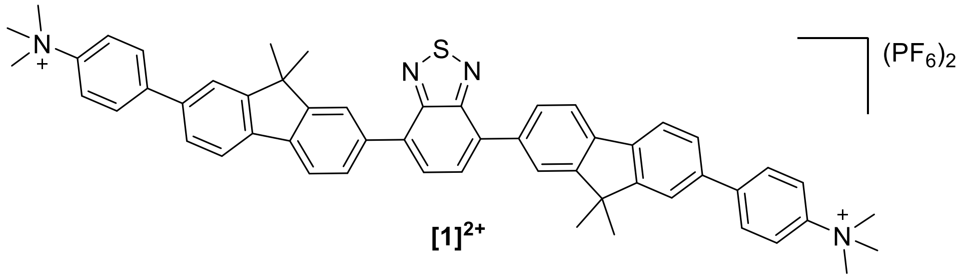

$[1]^{2+}$

In a pressure flask **afbfa** (100 mg, 0.13 mmol, 1.00 eq.) in acetonitrile (15 mL) was degassed with argon and excess methyl iodide (2 mL, 32.1 mmol, 244 eq.) was added. The mixture was heated to 90 °C for 24 h. After 24 h, the mixture turned deep yellow with some precipitate. The solution was filtered and dissolved in ethyl acetate and stirred for 30 min with 10% aqueous solution of ammonium

hexafluorophosphate. The precipitate was filtered and washed with ethyl acetate and dried under a vacuum. To purify the crude product, a silica column was used. The first eluent used was dichloromethane, to remove the first band. Later, the eluent was changed to acetonitrile: 1 M NH_4PF_6 (water) (4:1) to collect the orange band. The orange fraction was dried in rotary evaporator and recrystallized in the mixture of acetonitrile and water. The reaction gave compounds **[1]**$^{2+}$$(PF_6)_2$ as yellow powder (0.11 g, 0.10 mmol) in 77% yield.

UV-vis (MeCN): λ_{max} = 330 nm and 410 nm.

Luminescence (MeCN) λ_{ex} at 410 nm: λ_{max} = 555 nm.

^{1}H NMR (400 MHz, *d*-MeCN) δ 8.20 (d, *J* = 1.1 Hz, 2H), 8.08 (dd, *J* = 7.9, 1.6 Hz, 2H), 8.00 (m, 10H), 7.90 (d, *J* = 1.3 Hz, 2H), 7.89 – 7.85 (m, 4H), 7.76 (dd, *J* = 7.9, 1.7 Hz, 2H), 3.59 (s, 18H), 1.64 (s, 6H).

MALDI-MS: calcd. for $C_{54}H_{52}F_6N_4PS$ (m/z): 933.35, found: 933.35.

Anal. calcd.: $C_{54}H_{52}F_{12}N_4P2S$: C, 60.11; H, 4.86; N, 5.19, found C, 59.90; H, 4.79; N, 5.13.

NMR Spectra

4,7-tris[7-p-N, N, N-trimethyl-aniline-9,9-dimethylfluoren-2-yl]-2,1,3-benzothiadiazole dihexafluorophosphate $[1]^{2+}(PF_6)_2$

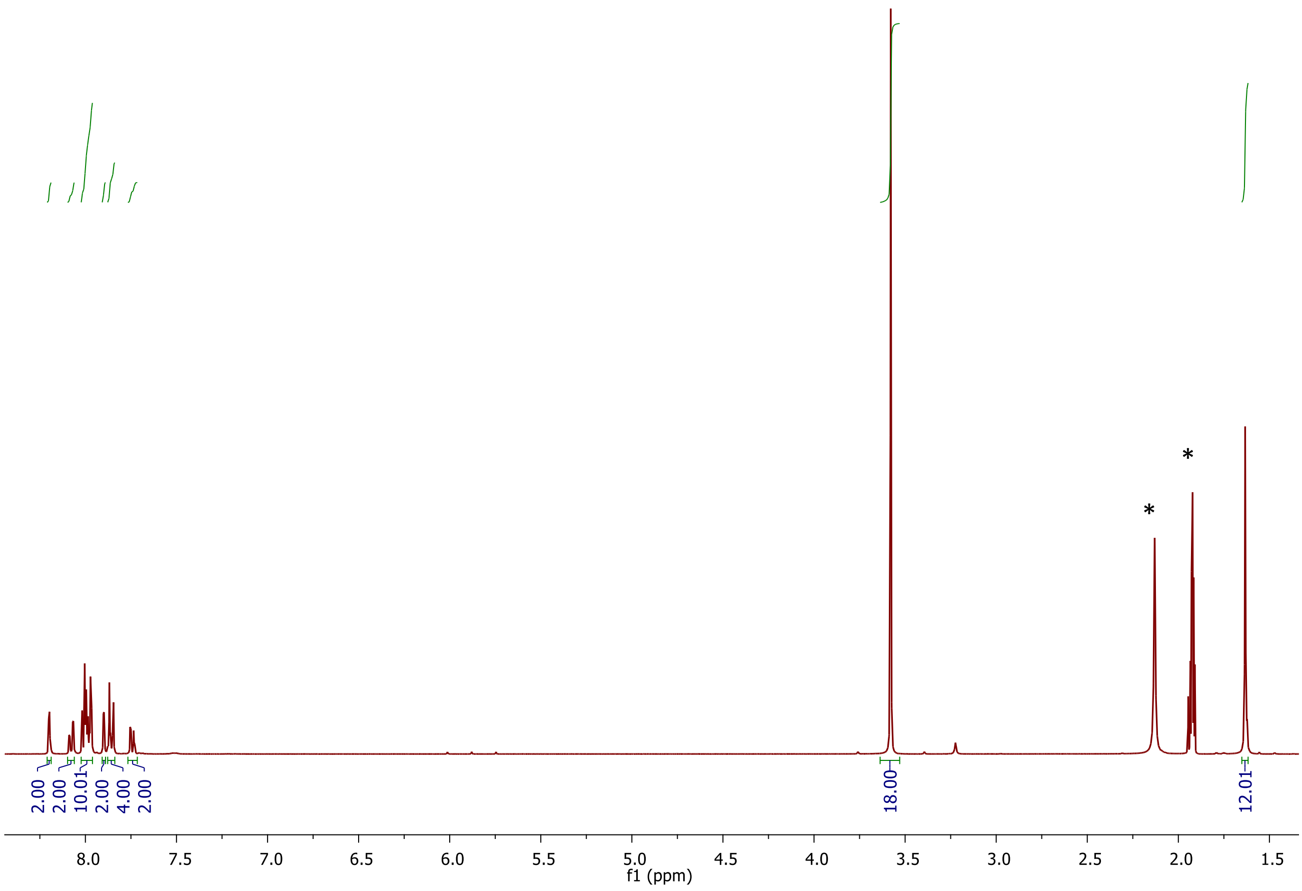

Figure. S2. ^{1}H-NMR spectrum of **[1]$^{2+}$**$(PF_6)_2$ in *d*-MeCN. The asterisk (*) indicates the residual solvent peaks of acetone and water.

4,7-bis[9,9-dimethylfluoren2-yl]-2,1,3-benzothiadiazole (fbf)

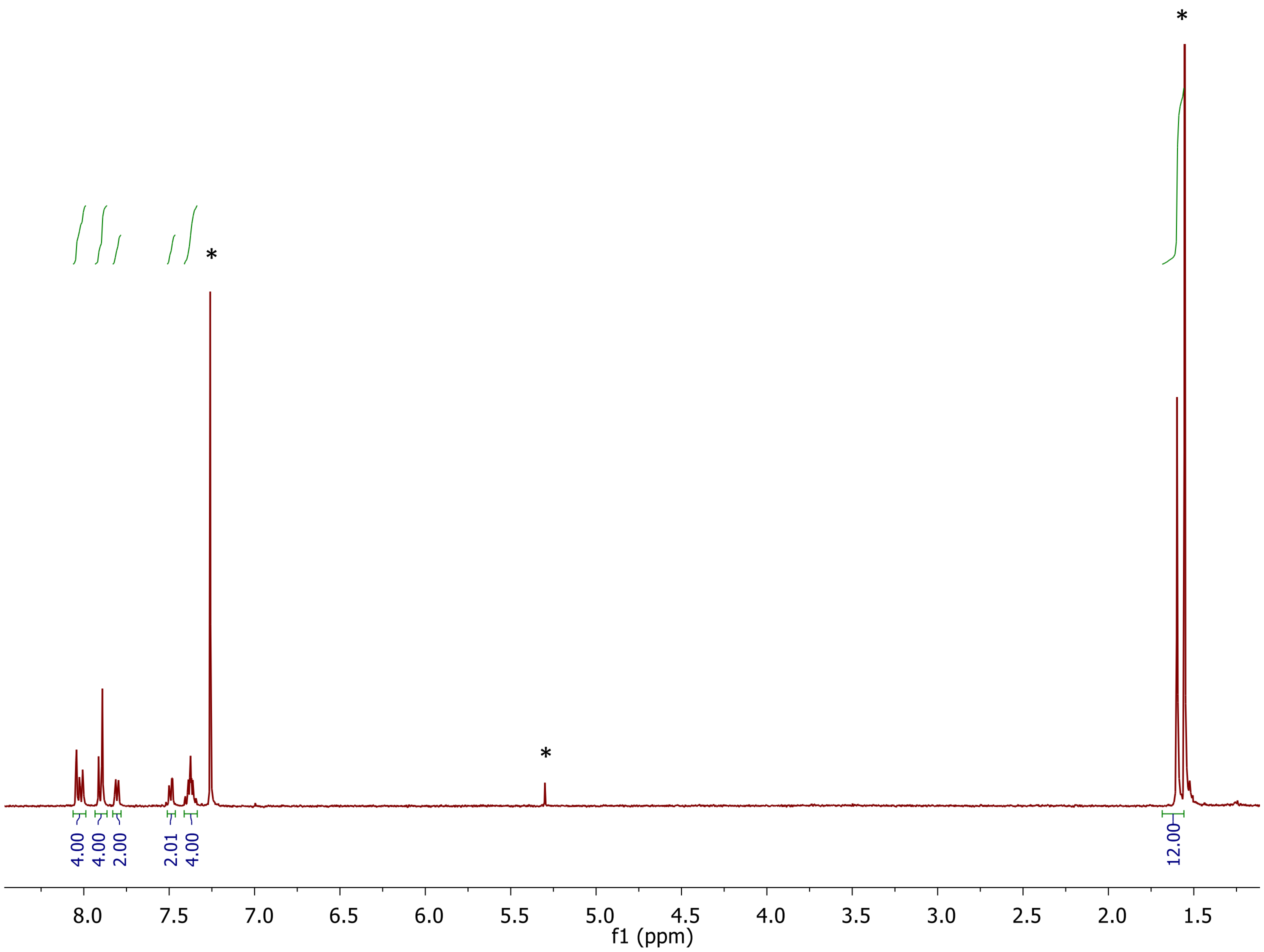

Figure. S3. ^{1}H-NMR spectrum of **fbf** in $CDCl_3$. The asterisk (*) indicates the residual solvent peaks of chloroform, water, and dichloromethane.

Crystal structure of $[1]^{2+}$

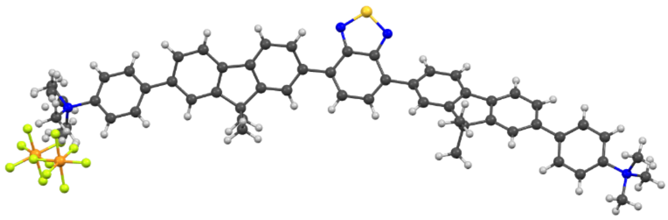

CCDC #2376492

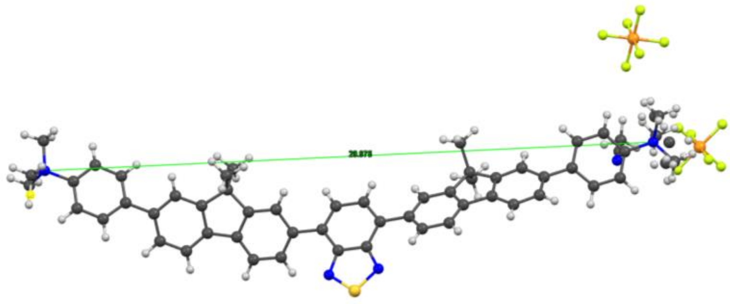

Distance between two terminal nitrogens is 2.99 nm.

Table 1 Crystal data and structure refinement for **[1]**$^{2+}$.

Identification code	NS23032_W7
Empirical formula	$C_{56}H_{55}F_{12}N_5P_2S$
Formula weight	1120.05
Temperature/K	150.0
Crystal system	monoclinic
Space group	C2/c
a/Å	30.244(2)
b/Å	12.2752(7)
c/Å	31.717(2)
α/°	90
β/°	116.043(4)
γ/°	90
Volume/Å^3	10579.5(12)
Z	8
ρ_{calc}g/cm^3	1.406
μ/mm^{-1}	0.208

F(000)	4640.0
Crystal size/mm^3	? × ? × ?
Radiation	MoKα (λ = 0.71073)
2Θ range for data collection/°	3.64 to 55.226
Index ranges	-39 ≤ h ≤ 39, -15 ≤ k ≤ 15, -41 ≤ l ≤ 41
Reflections collected	114845
Independent reflections	12215 [R_{int} = 0.0916, R_{sigma} = 0.0410]
Data/restraints/parameters	12215/0/726
Goodness-of-fit on F^2	1.082
Final R indexes [I>=2σ (I)]	R_1 = 0.0727, wR_2 = 0.1692
Final R indexes [all data]	R_1 = 0.0971, wR_2 = 0.1811
Largest diff. peak/hole / e Å^{-3}	0.39/-0.63

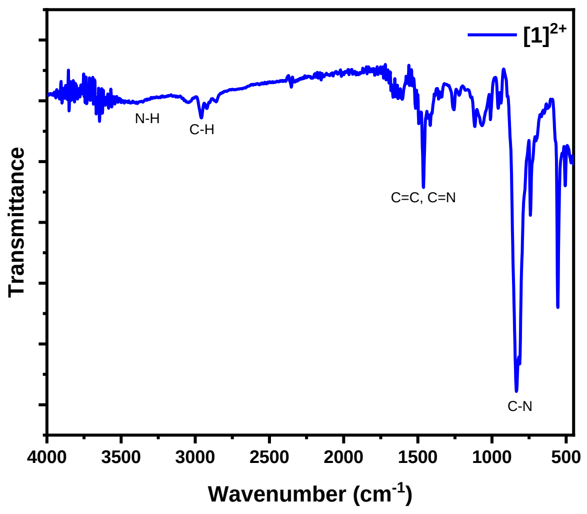

Figure. S4. IR spectrum of **[1]**$^{2+}$

S2. Liposome preparation

Stock solutions. The lipids 1,2-dipalmitoyl-sn-glycero-3-phosphocholine (DPPC) and 1,2-dimyristoyl-sn-glycero-3-phosphoethanolamine-N-[methoxy(polyethylene glycol)-2000] (ammonium salt) (14:0 PEG2000 PE) were prepared as stock solution in $CHCl_3$ with the concentration of 5 mM and 0.05 mM respectively. The chromophore **[1]**$^{2+}$$(PF_6)_2$ salt was prepared with a concentration of 0.05 mM in acetonitrile. Phosphate buffer pH 7.0 was prepared with the mixture of KH_2PO_4 (626 mg, 4.6 mmol), $K_2HPO_4 \cdot 3H_2O$ (1.186 g, 5.2 mmol), and K_2SO_4 (1.70 g, 9.74 mmol) in Milli-Q water (1 L) to reach a final pH of 7.0. The tetrazolium dye XTT (sodium;4-methoxy-5-[3-(2-methoxy-4-nitro-5 sulfonatophenyl)-5-(phenylcarbamoyl)tetrazol-3-ium-2-yl]-2 nitrobenzenesulfonate) and NADH (nicotinamide adenine dinucleotide) were prepared with a concentration of 1 mM in phosphate buffer pH 7.0.

Preparation and characterization of giant vesicles and liposomes. Giant vesicle and liposomes were prepared as follows. To prepare lipid film, in a 5 mL round bottom flask, 1 mL stock solution of DPPC (5.0 mM) and 1 mL 14:0 PEG2000 PE (0.05 mM) in $CHCl_3$ and 1 mL **[1]**$^{2+}$ (0.05) in acetonitrile were combined. The organic solvents were evaporated under vacuum leading to deposition of lipid film on the flask wall. The lipid film was dried under high vacuum for at least one hour and hydrated with phosphate buffer pH of 7.0 and 15 mg of NADH. The dispersed lipid film was repeatedly freeze-thawing, using liquid N_2 and a water bath at 52 °C yielding giant vesicles.

Prior to microscopy, and if applicable, giant vesicles were subjected a Sephadex G-25 size exclusion chromatography (SEC) column (6 cm length, 2 cm diameter) using phosphate buffer pH 7.0 as eluent. To prepare the sample for confocal microscopy measurements, a 50-100 μL amount of the giant vesicle sample was loaded into a well at the sample wells. Next, 200 μL of a freshly prepared, air-cooled agarose solution (1 weight-% in water) was added to the well and mixed with the sample. The mixed solution was then allowed to settle at room temperature before confocal microscopy measurements were taken.

To obtain liposomes of uniform size, the dispersion was extruded at 52 °C through 200 nm cellulose membrane filters 11 times with an Avanti Polar Lipids mini-extruder. The mixture of liposomes was treated with an SEC column, with phosphate buffer pH 7.0 as eluent, to separate the unencapsulated NADH. The liposome solution was collected and measured using dynamic light scattering (DLS). The results showed a Z_{Avg}-diameter ranging from 110-140 nm with a polydispersity index of about 0.1.

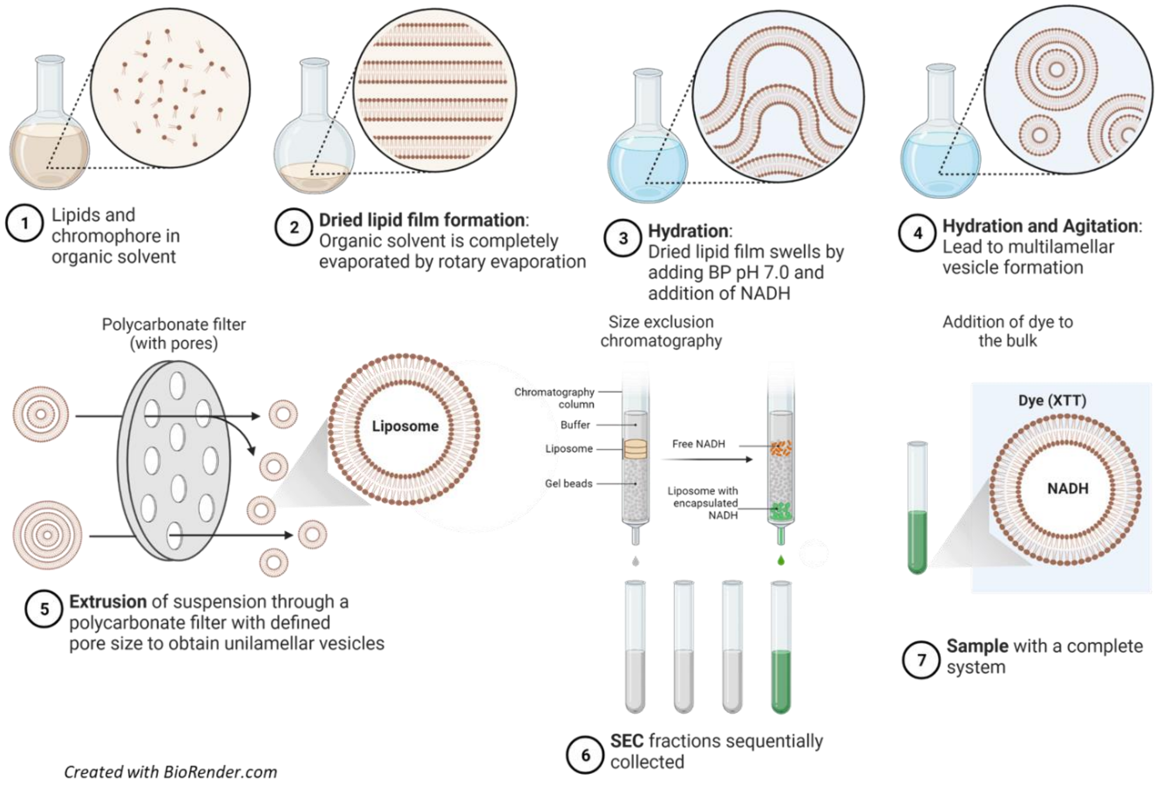

Figure. S5. Liposome preparation via thin film hydration with NADH encapsulated in the inner part of liposomes. Created with Biorender.com.

S3. The typical hydrodynamic diameter of DPPC liposome containing $[1]^{2+}$

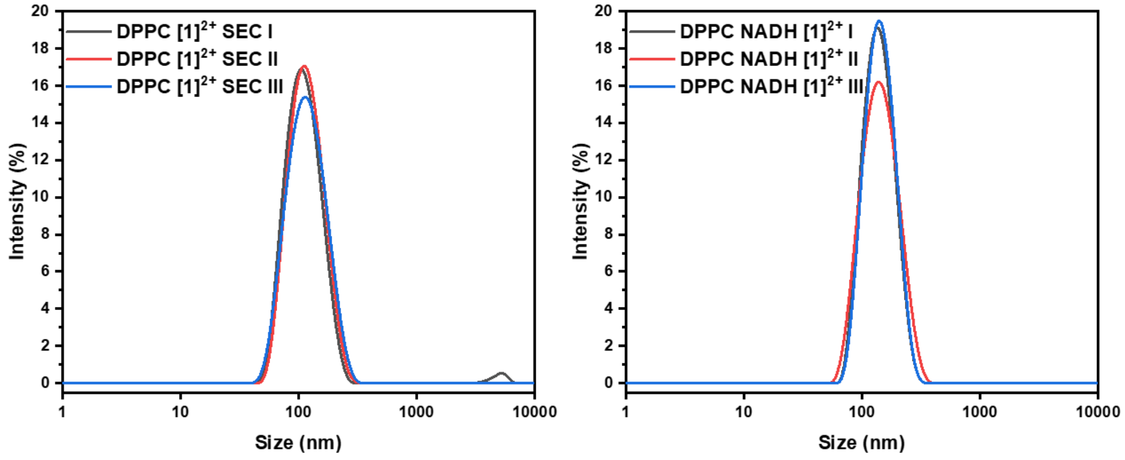

Figure. S6. Typical DLS measurements of DPPC:(14:0 PEG2000 PE):**[1]**$^{2+}$= 100:1:1 liposomes without NADH (left) and with encapsulated NADH (right).

Table S1. DLS data: average size and polydispersity index, before and after irradiation of samples.

Parameters	DPPC **[1]**$^{2+}$		DPPC NADH: **[1]**$^{2+}$	
	Before	After	Before	After
Z-average (nm)	108	109	134	137
Polydispersity index	0.12	0.13	0.08	0.08

S4. Lifetime of [1]$^{2+}$ in different environments.

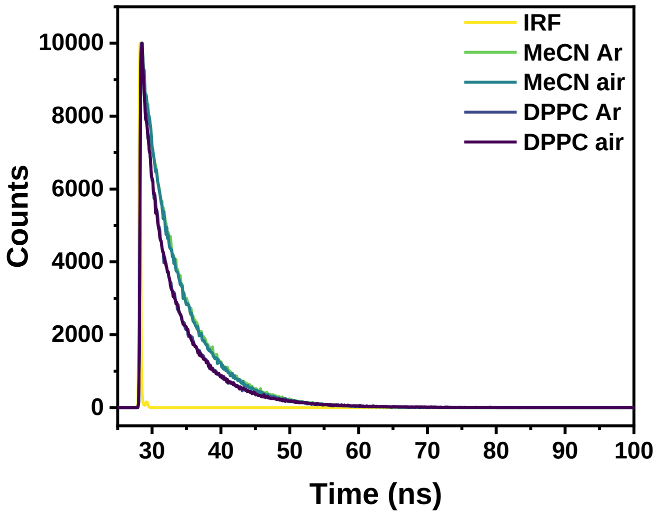

Figure S7**.** Lifetime measurements of **[1]**$^{2+}$ in acetonitrile (MeCN) and DPPC liposome under inert and ambient air atmosphere. IRF = instrument response function.

S5. Computational details for the computation of electronic excited states and Fukui functions

All quantum chemical computations were performed with the software Gaussian 16.[1] In order to properly describe the photophysical properties of **[1]**$^{2+}$, we benchmarked the performance of five functionals against the experimental absorption maxima. These functionals include the popular hybrid functionals B3LYP,[2–4] and PBE0[5–7] (PBE1PBE in Gaussian 16), and, because we expect **[1]**$^{2+}$ to find delocalized and possibly charge-transfer excitations, three functionals including long-range corrections: CAM-B3LYP,[8] which is based on B3LYP, both the original[9–11] and the revised[12] versions of lc-ωPBE, which use the same exchange-correlation functional as PBE0, and the wB97XD[13] functional by Head-Gordon and coworkers. Based on obtained results, we modified the HF exchange of the two most promising two functionals, PBE0 and CAM-B3LYP, to better reproduce the experimental excitation energies. Specifically, we increased the HF exchange in PBE0 from the default 25% to 30%

and denote this adapted functional as PBE0-30. Additionally, we reduced the μ-parameter in the error function describing the attenuation of CAM-B3LYP from the 0.33 in the default implementation to 0.20 and 0.15, denoted as CAM-B3LYP-20 and CAM-B3LYP-15, respectively. Dispersion was corrected for empirically using Grimme's D3 model with Becke-Johnson damping[14] except for wB97XD, which is already corrected for dispersion effects. These five functionals were tested in combination with a smaller def2-SVP and a larger def2-TZVP basis set,[15,16] but since the larger basis set barely affects the energetic position and intensities of the excitations, we deem the smaller basis set sufficiently accurate. Solvent effects for acetonitrile were included implicitly using a conductor-like polarization continuum model.[17,18] For each functional, **[1]**$^{2+}$ was optimized, and the convergence to a minimum was confirmed by the absence of imaginary frequencies within the harmonic approximation. The xyz-coordinates of the final structure can be found in section S14. Thirty singlet excited states were computed using time-dependent density functional theory for each of the eight functional/basis set combinations. The transition energies and oscillator strengths for the S_1 and S_3 excitations are shown in Figure S8.

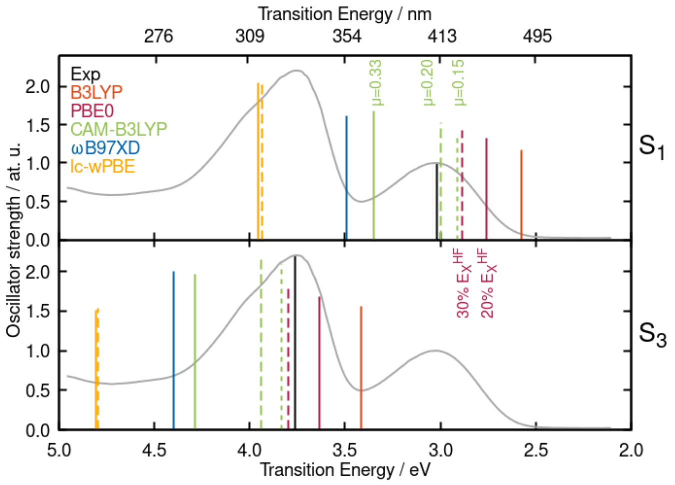

Figure S8**.** Vertical excitations for the tested functionals (see legend). The solid lines indicate the default implementation of the functionals, while dashed lines represent modified versions of the functionals with labels indicating the differences. For lc-ωPBE, the solid line represents the revised version (also known as lc-ωHPBE), and the dashed line the original implementation. Experimentally recorded band peaks are indicated as black lines, and the experimental spectrum is superimposed in grey.

Both for the S1 and S3 excitations, the excitations are strictly ordered by the HF exchange included in the exchange correlation functional, with increasing HF character correlated to an increased transition energy. This behaviour is well documented in literature.[19,20] One should note that the HF exchange in hybrid functionals and the error function parameters in range-separated functionals are not directly

comparable, but, in general, range separated functionals have a higher HF exchange that their local hybrid counerparts. The default implementations of PBE0 and CAM-B3LYP exhibit the most accurate excitation energies with respect to the experiment of all the traditional implementations, which is why we opted to modify these. Since PBE0 underestimates the excitation energies for both excitations, we increased the HF character; by contrast, CAM-B3LYP overestimates these, which is why we reduced the µ-parameter. The modified versions greatly improve the results, even if all slightly underestimate the excitation energy of the transition to S_1 and overestimate that to the S_3. In order to incorporate the long-range effects included in CAM-B3LYP (and its modified versions), we excluded PBE0-30 from further consideration and computed ensemble absorption spectra using CAM-B3LYP-20 and CAM-B3LYP-15 from Wigner-sampled geometries. We generated these structures with the workflow implemented in the SHARC[21] program package based on a harmonic frequency calculation with each of the functionals. The ensemble was generated at a temperature of 300 K and comprised 20 geometries. To ensure convergence, we limited the sampling to normal modes above 480 cm^{-1}. The convoluted spectra are shown in Figure S9.

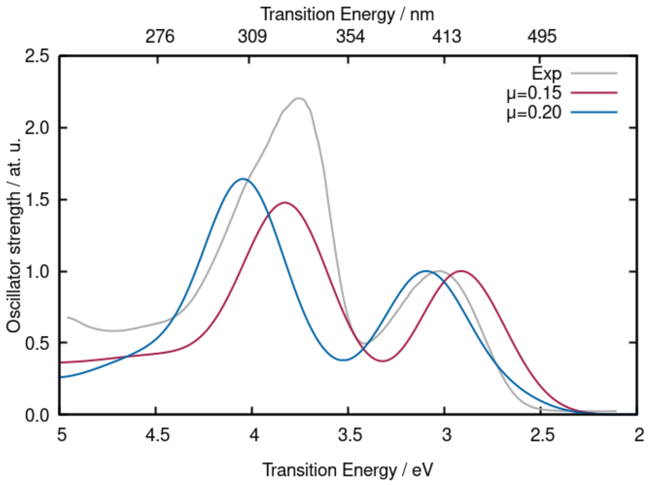

Figure S9**.** Absorption spectra computed with CAM-B3LYP-20 (blue) and CAM-B3LYP-15 (red) from the Wigner ensemble. The experimental spectrum is superimposed in grey. For comparison, all spectra were normalized at the S_1 peak to 1.

Based on the Wigner sampling, CAM-B3LYP-15 produces two peaks at 324 and 425 nm, and with CAM-B3LYP-20 the bands peak at 306 and 400 nm. While both modified versions of the CAM-B3LYP functional reproduce the experimental spectrum reasonably well, which peaks at 330 and 410 nm, respectively, CAM-B3LYP-20 reproduces best the S_3 energy and CAM-B3LYP-15 the S_1. Hence, we based our decision for what functional to use in the end on Koopmans theorem[22] by comparing the HOMO energies to the ionization energy. According to the theorem, the energy eigenvalue of the HOMO should equal the negative ionization energy, which we compute here as the difference in energy

between **[1]**$^{2+}$ and **[1]**$^{3+}$ (the molecule after ionization) at the same geometry. For CAM-B3LYP-15, the HOMO energy is about half the negative ionization energy (-0.26 vs. 0.50 at. units), while for CAM-B3LYP-20, the energies are in good agreement (-0.26 vs. 0.23 at. units). Therefore, we opted for CAM-B3LYP-20/def2-SVP for all analyses presented in the main manuscript and the following section.

Fukui functions were computed on the optimized geometry of **[1]**$^{2+}$ by performing single point calculations with a total charge of either +1, +2 or +3. As we are interested in the electrochemical properties of **[1]**$^{2+}$ after light absorption, we need to compute the Fukui functions for the relevant excited state. According to Kasha's rule,[23] which says that any excited state populations relax to the lowest excited state of the respective multiplicity within a short period of time, we computed the Fukui functions for the S_1. The Fukui functions in the S_1 can in turn be approximated as the inverse of the ground state Fukui functions.[24] f^- was hence computed as the difference in electron density between charge +2 and +1, which corresponds to f^+ in the ground state. f^+ was analogously computed as the electron density between +2 and +1, corresponding to f^- in the ground state. Δf was computed as the difference between the two Fukui functions.

Visualization of the NTOs, HOMO and LUMO as well as the Fukui functions was done with the molecular viewer Jmol[25] with cutoff values set to 0.04 a.u. for the orbitals, and 0.02 a.u. for the Fukuifunctions. Excited state wave function analysis was performed with the TheoDORE[26] suite.

S6. Characterization of the excited states

Both bright S_1 and S_3 excited states of **[1]**$^{2+}$ are of ππ* character, as evidenced by the natural transition orbitals (NTOs), shown in **Figure 3e-f** in the main manuscript**.** NTOs are orbitals representing the excitations in terms of the excited electron and the ground-state hole and generally provide a better description of the transition compared to standard molecular orbitals. During the excitation to the S_1 (**Figure 3e**), electron density is transferred from the five six-membered rings closest to the center of the molecule to the most central moiety. This leads to a sizeable increase in the static dipole moment to 11.1 Debye for the S_1 compared to 7.5 Debye in the electronic ground state S_0. By contrast, in the S_3 (**Figure 3f**), both the hole and the excited state electron are similarly delocalized over large parts of the molecule, making the S_3 comparable in electronic properties to the ground state, with the dipole moment only moderately increasing to 9.2 Debye.

The transition dipole moments for the excitation to the S_1 and the S_3 are shown in Figure S10. Both are reasonably well aligned along the molecular wire and confirm that a single **[1]**$^{2+}$ absorbs radiation above 300 nm along this axis.

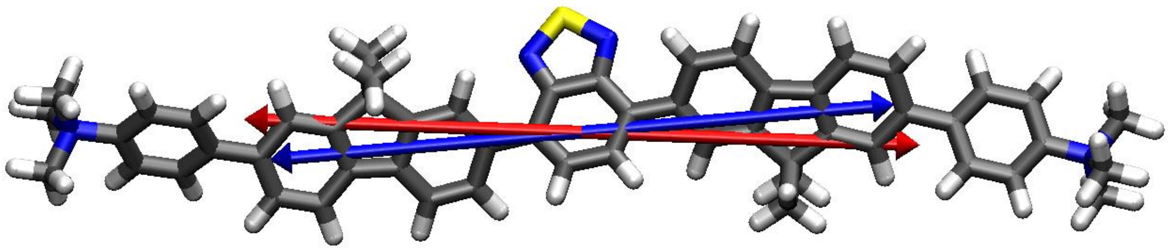

Figure S10. Transition dipole moments for the S_1 (blue) and S_3 (red) excitations.

As stated above, the S_3 exhibits an electronic character that is closer to that of the ground state compared to the S_1, which is of a higher charge transfer character. To support this claim, we divided **[1]**$^{2+}$ into six fragments (see Figure S11a) and computed the charge transfer matrix between these states for the S_1 and S_3 excitations (panels b and c of the same figure).

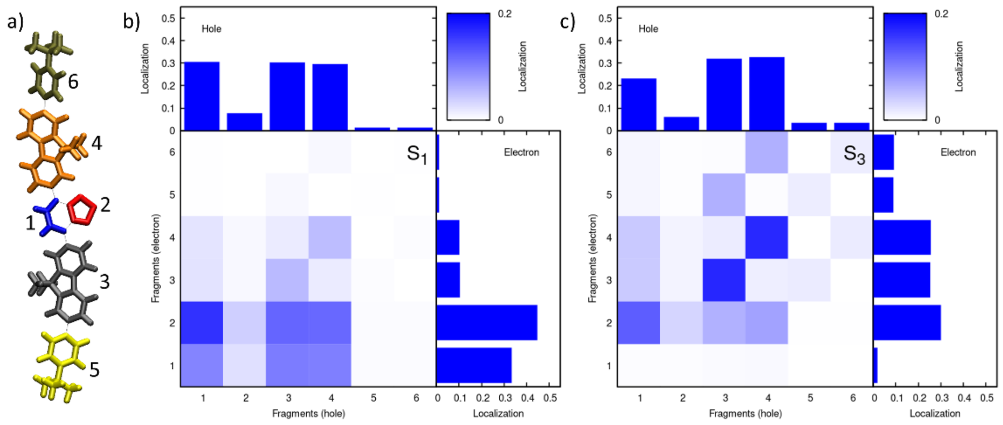

Figure S11. a) Definition of fragments. b) and c) Charge transfer matrix for the excitations to S_1 and S_3., respectively.

The S_1 exhibits multiple charge transfer elements, such as from the six- to the five-membered ring of the central moiety (fragments 1 and 2, respectively), as well as to these two fragments from the adjacent fragments 3 and 4. This means that in the S_1, the five-membered ring hosts significantly more electron density compared to the S_0. In contrast, the excitation to S_3 is more delocalized over the entire molecule, with all fragments except fragment 1 contributing notably. However, the largest weights are on the diagonal and correspond to local excitations on fragments 3 and 4. Since this local character dominates, the resulting electrostatic distribution in the S_3 is more similar to that of the S_0, which can also be seen from the electrostatic potential (ESP) maps in Figure S12.

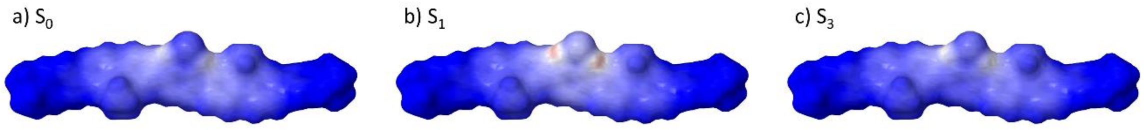

Figure S12. Electrostatic potential of the S_0, S_1 and S_3 states mapped onto a van der Waals surface. Blue areas indicate positive ESPs, white neutral and red negative areas. Blue saturation is where the ESP exceeds a partial charge of +0.15.

In all three states, the ends of **[1]**$^{2+}$ exhibit a mostly positive ESP due to the positive charge on the methylammonium groups. This positive charge extends over the entire molecule, but the central rings are more on the neutral side. Only the S_1 exhibits regions with a net-negative ESP due to the increased electron density on fragment 2. The central moiety of the S_3 is slightly less negative than the S_0, but much less so than the S_1. This similarity in electronic structure between S_0 and S_3, and the fact that the S_1 is quite different from the other two, explains why the excitation to the S_1 is red shifted in DPPC compared to in acetonitrile, while the excitation to S_3 is barely affected.

S7. Confocal microscopy

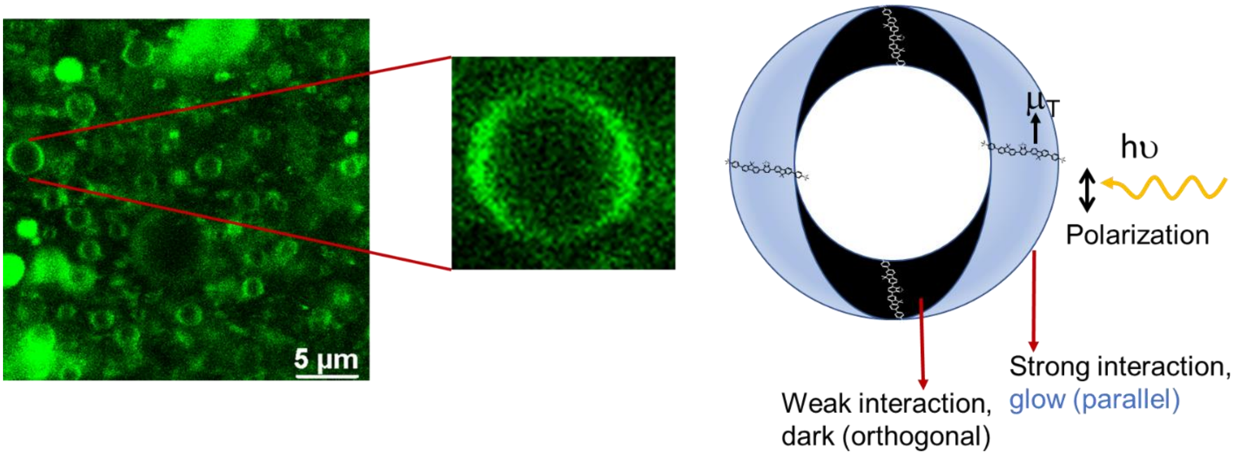

Figure S13. Confocal luminescence microscopy image

The confocal luminescence microscopy image displays a double-half moon shape that results from the interaction of **[1]**$^{2+}$ with the laser beam's incident light. When the laser beam moves parallel to the transition dipole moments of **[1]**$^{2+}$, a strong interaction occurs, leading to high emission intensity. On the other hand, when the laser beam moves orthogonally, a weak interaction happens, leading to less emission. The transition dipole moments of **[1]**$^{2+}$ are aligned along the membrane. The double-half moon effect reveals the preferred orientation of **[1]**$^{2+}$ in the lipid bilayer, but it cannot distinguish between parallel or orthogonal alignment. The preferred arrangement has been unraveled using molecular dynamics (MD) simulations; see below.

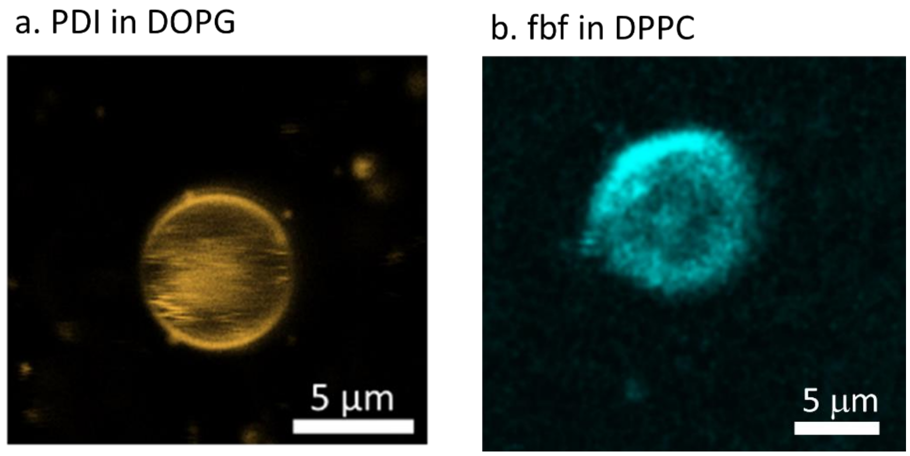

Figure S14. Confocal luminescence microscopy image, a) our previously reported system (reproduced from Ref. 27, with permission from The Royal Society of Chemistry based on a CC BY 3.0 license) with a preferably parallel orientation of the dye,[27] and b) the image of the reference compound fbf in the DPPC liposome.

In another reported work from us, the respective dye aligned alignment mostly parallel to the membrane surface and produced a 90° different half-moon-shape with the same laser alignment (Figure S14a).[27]

Furthermore, the hydrophobic core unit of **[1]**$^{2+}$ was integrated into the DPPC lipid bilayers (Figure S14b) and no half-moon effect was observed, which is concluded to be due to a random orientation within the lipophilic part of the membrane.

S8. Computational details on the membrane assembly simulations

Classical force field MD simulations of the lipid bilayer assembly were carried out to elucidate the orientation and alignment of **[1]**$^{2+}$. To this aim, we randomly distributed 128 DPPC lipids and one molecule of **[1]**$^{2+}$ in a cubic box of 70 Å side length. Due to the low concentration in the experiment, we did not include any 14:0 PEG2000 PE molecules in the simulation. The remaining box volume was then filled with water molecules and KCl ions to ensure charge neutrality and attain a salt concentration of 0.15 mol l^{-1}. The starting systems were assembled with the input generator CHARMM-GUI.[28]

All MD simulations were performed using the program packages Amber22 and Ambertools22.[29] **[1]**$^{2+}$ was described using the generalized Amber force field included in AmberTools22, with ground state point charges computed within the restrained electrostatic potential atomic partial charge (RESP) scheme in antechamber (part of Ambertools22) based on electrostatic potential date

computed in Gaussian16 on the B3LYP/def2-SVP level of theory (see above). The lipids were described using the Lipid21 force field,[30] while water and the atomic ions were described using the "optimal" point charge model[31] implemented in AmberTools22. After a 10.000 step minimization with 5000 steps employing the steepest gradient algorithm and another 5000 steps using a conjugate gradient algorithm, the systems were heated using a Langevin thermostat at a collision frequency of 1.0 ps^{-1} to 100 K in 2500 time steps (5 ps), followed by heating to 300 K in 50 000 steps (100 ps). Finally, the systems were propagated until the membrane was assembled and all water pores had disappeared. The insertion mode of **[1]**$^{2+}$ was then investigated by eye inspection.

For all simulations, a time step of 2 fs was used with the SHAKE algorithm[32] applied to freeze hydrogen bonds at a relative geometric tolerance of $1*10^{-7}$, thus enabling large time steps. Constant pressure periodic boundary conditions were superimposed with anisotropic pressure scaling control with Berendsen[33] at a pressure relaxation time of 1 ps, which was increased to 2 ps for the second heating phase only. The cutoff for non-bonded interaction terms was set to 10 Å. The simulations were performed using the GPU (CUDA) version of pmemd.[34–36] Visualization was done with VMD.[37]

The final points of the eight trajectories are shown in Figure S15. In all eight cases, as noted in the main manuscript, **[1]**$^{2+}$ is arranged in a transmembrane fashion. We note, however, that this configuration impacts the membrane topology, as in five out of the eight simulations, the membrane is visibly distorted where **[1]**$^{2+}$ is embedded (see **Fig. 4 a**, **c**, **f**, **g**, and **h**). It seems like **[1]**$^{2+}$ is slightly too short for an ideal integration into the membrane, causing in the membrane surface to be wavy to reduce its width at this position. This distortion of the membrane is visible in most of the trajectories at some points along the simulation, but in a few cases, the membrane becomes smooth during the simulated timeframe (see the eight trajectory movies as part of this SI). Thus, we believe that the distortion of the membrane is a computational artifact that can be removed when simulating higher time scales.

Our assessment of the possibility of membrane deformation within the context of MD by extending the simulation duration may not yield additional substantive. As such, deformation is a natural aspect of the dynamic process involved; as for the stability of the wires in the membrane, we can observe in our simulations that the orientation only changes as long as there are water pores present in the membrane, which have all evaporated at the end of our trajectories. As the molecular wire orients transmembrane in all simulations, once the pores are gone, we can say that this is the direction the wire will orient.

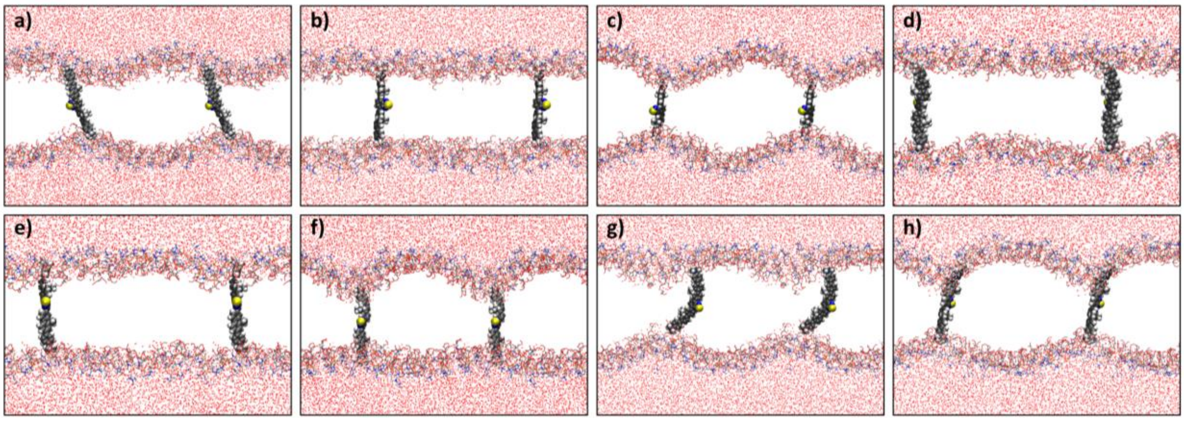

Figure S15. (a-h) Final point of trajectories after 0.5-1 μs, showing how **[1]**$^{2+}$ orients in the membrane. Each graphic shows two periodic images of **[1]**$^{2+}$. Color code: red = oxygen, white = hydrogen, blue = nitrogen, yellow = sulfur, golden = phosphorus, and grey = carbon. The alkyl tails of the lipid bilayer are omitted for clarity.

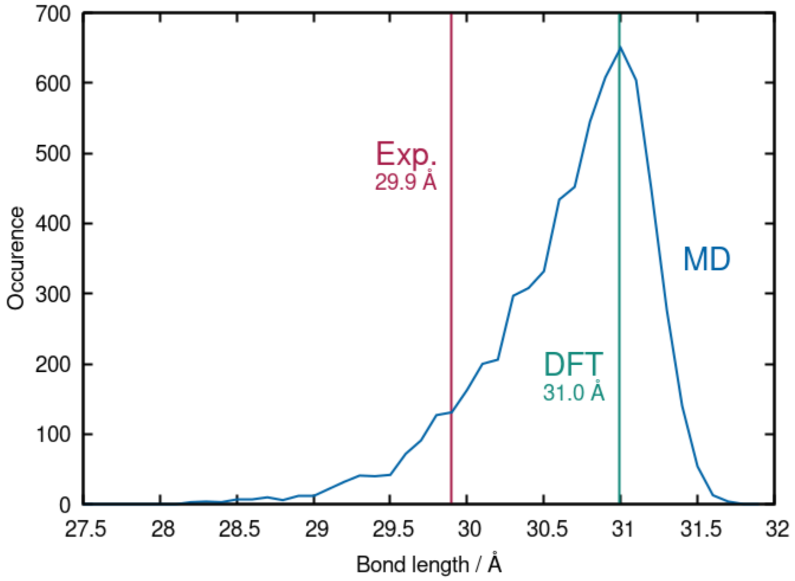

Figure S16. The distance of two $N(CH_3)_3$-groups at **[1]**$^{2+}$. The red line is based on the crystal structure, the green line is from the DFT calculation, and the distribution distance from the MD simulation is from 2.8-3.15 nm.

S9. Calceine-luminescence leakage test

We conducted a calceine test to check the impermeability of the membrane, which is a standard procedure used to assess the potential for membrane disruption during encapsulation with slight modifications.[38,39] We observed the DPPC liposome with and without integration of **[1]**$^{2+}$ and monitored the solution over varying time frames, but did not detect any leakage. To determine if light

could induce membrane disruption, we analyzed the same sample after 2 hours of irradiation with 470 nm LED and still did not observe any calceine leakage.

The calceine test preparation is as follows: in a 5 mL round bottom flask, 1 mL stock solution of DPPC (5.0 mM), 1 mL 14:0 PEG2000 PE (0.05 mM) and 1 mL **[1]**$^{2+}$ (0.05 mM) in acetonitrile were combined. The organic solvents were evaporated under vacuum for at least one hour. Calceine (43.58 mg, 0.07 mmol) was added to the flask and hydrated with 1 mL phosphate buffer (K_2HPO_4/KH_2PO_4) pH 7.7 to yield a calceine concentration of 70 mM. The dispersed lipid film was freeze-thawed for three times, using liquid N_2 and a water bath at 52 °C. The dispersion was extruded at 52 °C through 200 nm cellulose membrane filters 11 times with an Avanti Polar Lipids mini-extruder. The obtained solutions of liposomes were separated from excess Calceine using a SEC with phosphate buffer pH 7.7 as eluent. Luminescence measurements were carried out with 10 μl of liposome solution diluted with 990 μl of phosphate buffer pH 7.7, monitored every 0, 1, 5, 10, 30, 60, 90, and 120 minutes, after the measurements, Triton X-100 (1 mM, 50 μl) was added to the liposome sample. The liposomes are disturbed by the addition of Triton X-100 and release the encapsulated calceine. To check the stability of encapsulation in liposomes, other samples were measured after 2 h irradiation with a 470 nm LED stick. All luminescence spectra were recorded from 495 – 700 nm with excitation at λ_{ex} = 485 nm. Experiments were carried out for 2 days to investigate the stability of membrane.

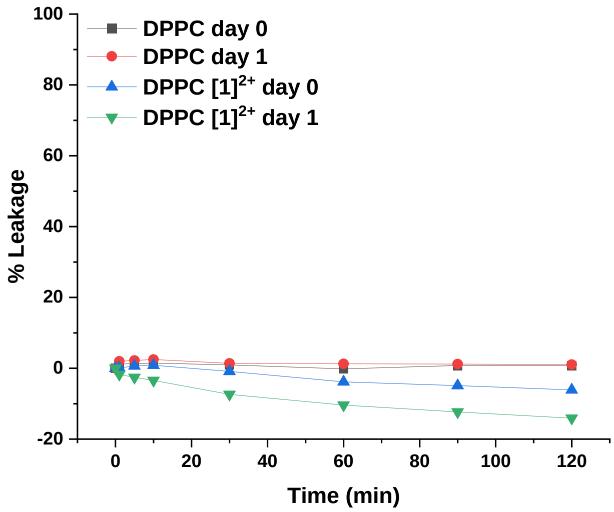

Figure S17. The percentage of calcein leakage is measured by comparing the luminescence before and after the addition of Triton X-100. This measurement is taken at λ_{max} = 518 nm, with and without the presence of **[1]**$^{2+}$.

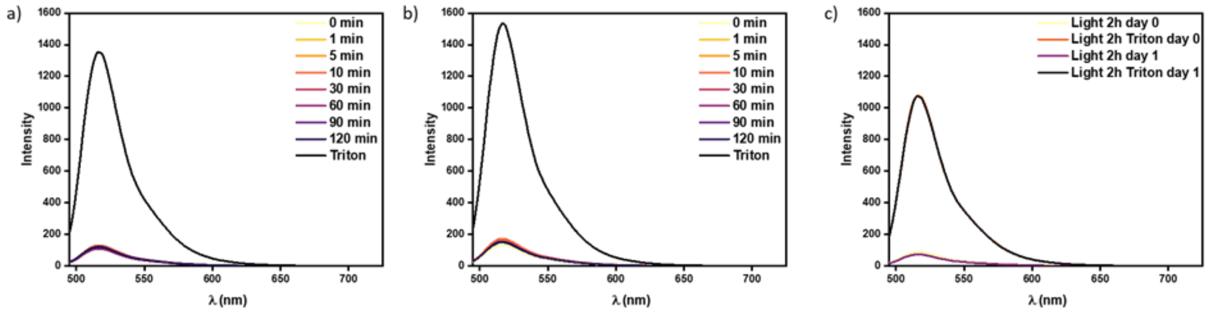

Figure S18. Luminescence spectra of calcein in DPPC liposomes at self-quenching concentration (low intensity) and spectra upon addition of Triton X-100 to disrupt the lipid membrane (high intensity), a) day 0, b) day 1, C) day 0 and 1 after irradiation with 470 nm LED stick for 2 h.

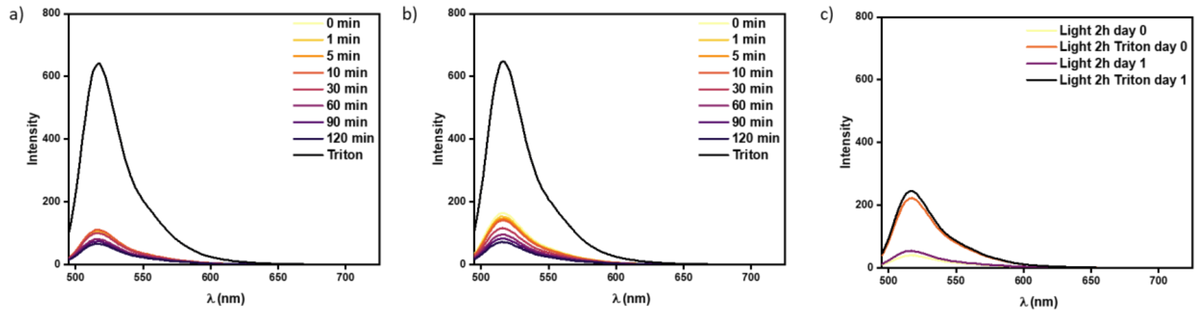

Figure S19. Luminescence spectra of calcein in DPPC liposomes with 1% **[1]**$^{2+}$ at the self-quenching concentration (low intensity) and spectra upon addition of Triton X-100 to disrupt the lipid membrane (high intensity), a) day 0, b) day 1, C) day 0 and 1 after irradiation with 470 nm LED stick for 2 h.

S10. Electrochemical and Spectroelectrochemical characterization

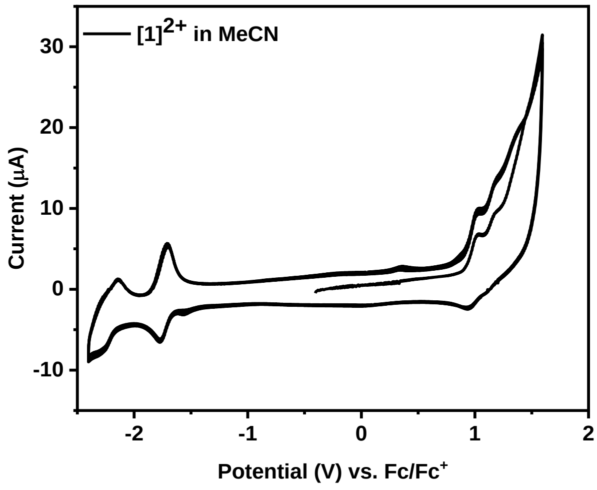

Figure S20. Cyclic voltammograms of **[1]$^{2+}$** (acetonitrile, 0.1 M nBu_4NPF_6 supporting electrolyte, 0.25 mM analyte, 100 mV s^{-1}, glassy carbon-working electrode, Pt-wire counter electrode, Ag wire (in a glass frit containing electrolyte solution-quasi reference electrode) referenced against Fc/Fc^+.

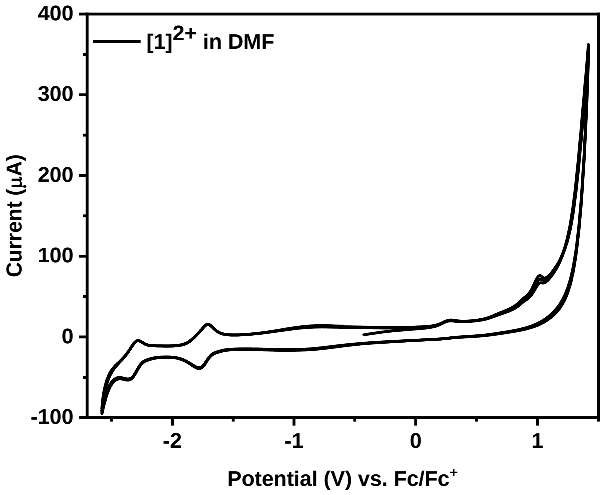

Figure S21. Cyclic voltammograms of **[1]$^{2+}$** (DMF, 0.1 M nBu_4NPF_6 supporting electrolyte, 0.25 mM analyte, 100 mV s^{-1}, glassy carbon-working electrode, Pt-wire counter electrode, Ag wire (in a glass frit containing electrolyte solution-quasi reference electrode) referenced against Fc/Fc^+.

Table S2. Summarized electrochemical data of **[1]$^{2+}$** in acetonitrile and DMF referenced against Fc/Fc^+ (potentials are accurate to $\pm$ 0.1 V), (Ox: Oxidation, Red: Reduction).

**[1]$^{2+}$**	1st Ox	2nd Ox	1st Red	2nd Red
Acetonitrile	1.0	1.2	-1.7	-2.2
DMF	0.3	-	-1.7	-2.3

The spectroelectrochemistry measurements were performed using a pine honeycomb screen-printed platinum electrode as a working electrode and counter electrode and Ag wire (in a glass frit containing electrolyte solution) as a quasi-reference electrode.

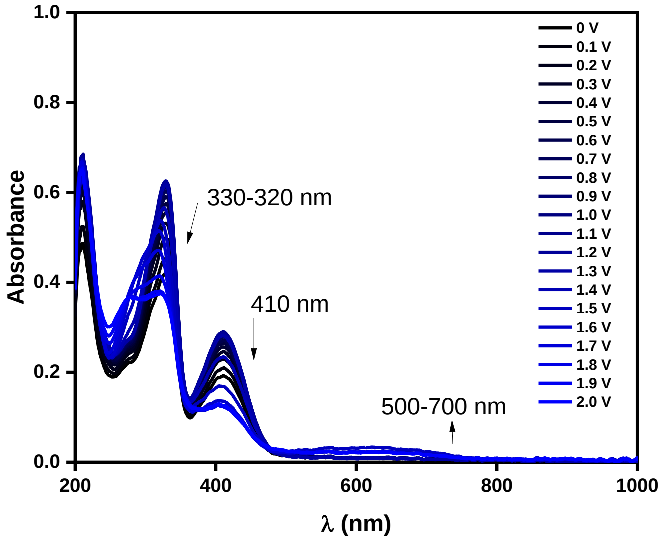

Figure S22. UV-Vis-NIR spectra obtained during spectroelectrochemical oxidation of **[1]**$^{2+}$ in deaerated acetonitrile containing 0.1 M nBu_4PF_6, measured from 0.0 V to 2.0 V, are given in V vs. Fc+ /Fc.

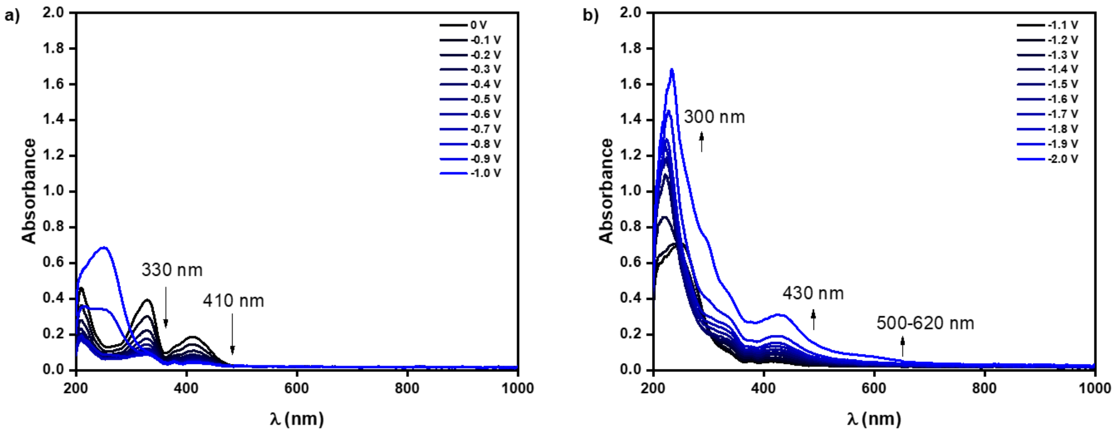

Figure S23. UV-Vis-NIR spectra obtained during spectroelectrochemical reduction of **[1]**$^{2+}$ in deaerated acetonitrile containing 0.1 M nBu_4PF_6, measured from 0.0 V to -2.0 V, are given in V vs. Fc+ /Fc.

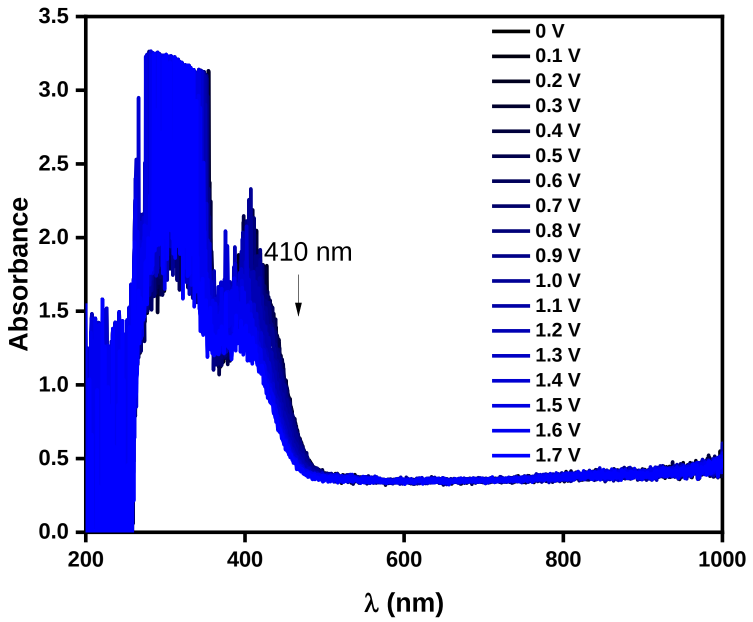

Figure S24. UV-Vis-NIR spectra obtained during spectroelectrochemical oxidation of **[1]**$^{2+}$ in deaerated DMF containing 0.1 M nBu_4PF_6, measured from 0.0 V to 1.7 V, are given in V vs. Fc+ /Fc.

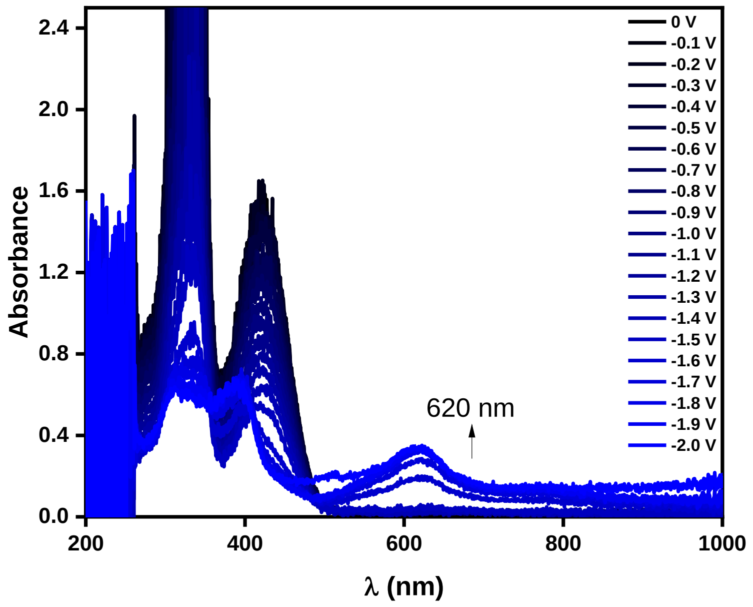

Figure S25. UV-Vis-NIR spectra obtained during spectroelectrochemical reduction of **[1]**$^{2+}$ in deaerated DMF containing 0.1 M nBu_4PF_6, measured from 0.0 V to -2.0 V, are given in V vs. Fc+ /Fc.

Thermochemistry of electron transfer from the photoexcited [1]$^{2+}$ followed by the oxidation of NADH and the reduction of XTT.

The potential energy of every component (vs SCE):

<u>1. **[1]**$^{2+}$ (measured in acetonitrile)</u>

Emission: 550 nm (r.t) based on this number, we determined the E_{00} of **[1]**$^{2+}$

$E_{00} = 1240/\lambda_{em} = 1240/550 = 2.3$ eV

Electrochemical potential for **[1]**$^{2+}$ from Fc^+/Fc to SCE (+0.38 V vs SCE)[40]

[1]$^{3+}$/**[1]**$^{2+}$ = 1.0 V vs Fc^+/Fc → 1.38 V vs SCE

[1]$^{2+}$/**[1]**$^{+}$ = -1.7 V vs Fc^+/Fc → -1.32 V vs SCE

[1]$^{2+\bullet}$/***[1]**$^{2+}$ = 1.38 V – 2.3 eV = -0.92 V vs SCE

***[1]**$^{2+}$/**[1]**$^{2+\bullet}$= -1.32 + 2.3 eV = 0.98 V vs SCE

<u>2. XTT</u>

XTT^-/XTT = -0.19 V vs Ag/AgCl (based on MTT data)[41]

Converted from Ag/AgCl to SCE (-0.044 V vs SCE)[42]

XTT^-/XTT = -0.23 V vs SCE

<u>3. NADH</u>

$NADH^+$/NADH = 0.65 V vs Ag/AgCl[43]

Converted from Ag/AgCl to SCE (-0.044 V vs SCE)[42]

$NADH^+$/NADH = 0.61 V vs SCE

<u>4. Driving force for electron transfer from **[1]**$^{2+}$ to XTT:</u>

From ***[1]**$^{2+}$ (excited state) to XTT

ΔG_{eT} = (**[1]**$^{2+\bullet}$/***[1]**$^{2+}$) - (XTT^-/XTT) = -0.92 V – (-0.23 V) = **-0.69 V**

From **[1]**$^{+}$ (reduced ground state) to XTT

ΔG_{eT} = (**[1]**$^{2+}$/**[1]**$^{+}$) - (XTT^-/XTT) = -1.32 V – (-0.23 V) = **-1.09 V**

From **[1]**$^{2+}$ (ground state) to XTT

ΔG_{eT} = (**[1]**$^{3+}$/**[1]**$^{2+}$) - (XTT^-/XTT) = 1.38 V – (-0.23 V) = 1.61 V

<u>5. Driving force for electron transfer from NADH to **[1]**$^{2+}$</u>

From NADH to ***[1]**$^{2+}$ (excited state)

ΔG_{eT} = ($NADH^+$/NADH) - (***[1]**$^{2+}$/**[1]**$^{2+\bullet}$) = 0.61 V - 0.98 V = **-0.37 V**

From NADH to **[1]**$^{3+}$ (oxidized ground state)

ΔG_{eT} = ($NADH^+$/NADH) - (**[1]**$^{3+}$/**[1]**$^{2+}$) = 0.61 V – 1.38 V = **-0.77 V**

From NADH to **[1]**$^{2+}$ (ground state)

ΔG_{eT} = (NADH^{+}/NADH) - (**[1]**$^{2+}$/**[1]**$^{+}$) = 0.61 V – (-1.32 V) = 1.93 V

S11. Photoirradiation

The typical reactions were carried out within quartz cuvettes where 300 μl of liposome solution, 10 μl of 1 mM XTT, and 190 μl phosphate buffer pH 7.0 were added. The mixtures were illuminated within a custom-made reactor equipped with four ventilators to exclude heating of the samples and an LED-stick (Nichia Corporation; NSPB500AS) (λ = 470 ± 10 nm, 15.7 mW measured with a Hioki 3664 optical power meter set to 470 nm).[44] The photon flux (f) was determined using standard ferrioxalate actinometry with a value of $8.2{\cdot}10^{-8}$ Einstein·s^{-1}. The samples were monitored via UV-Vis spectroscopy after 0, 1, 5, 10, 30, 60, 90, and 120 minutes. The number of photons flux absorbed by **[1]**$^{2+}$ was determined by measuring the initial absorption at 470 nm. To calculate the number of photons (n_{photon}) over time, the photon flux from the initial absorption was multiplied by the average of the latest two irradiation times. The number of formazan (n_{fz}) (Fz) was calculated by measuring the increasing absorbance at 470 nm; the increasing absorbance value was converted to the number of Fz formed with the reported extinction coefficient of Fz at 470 nm (21.600 M^{-1} cm^{-1}).[45] The quantum yield (Qy) of Fz formation was calculated by dividing the number of Fz formed by the number of photons absorbed.

$$n_{photon} = f \times (\frac{t_0+t_1}{2}) \qquad \text{(S1)}$$

$$Qy = \frac{n_{fz}}{n_{photon}} \qquad \text{(S2)}$$

Equation S1 determines the number of photons during irradiation time. Equation S2 is used to calculate the quantum yield of formazan formation.

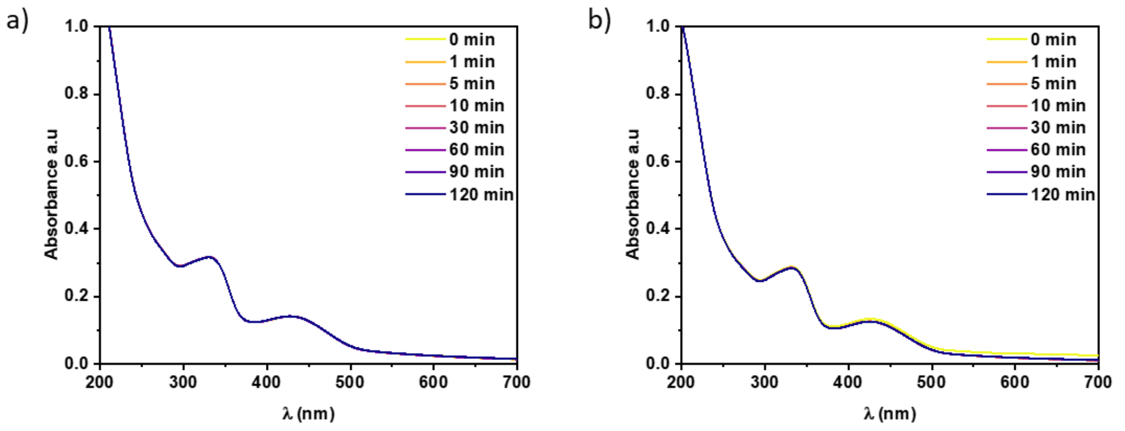

Figure S26. Temporal UV-vis spectra in DPPC:(14:0 PEG2000 PE):**[1]**$^{2+}$ = 100:1:1 (/**[1]**$^{2+}$/) liposome in phosphate buffer pH 7.0, irradiated with 470 nm LED stick a) ambient air; b) argon atmosphere.

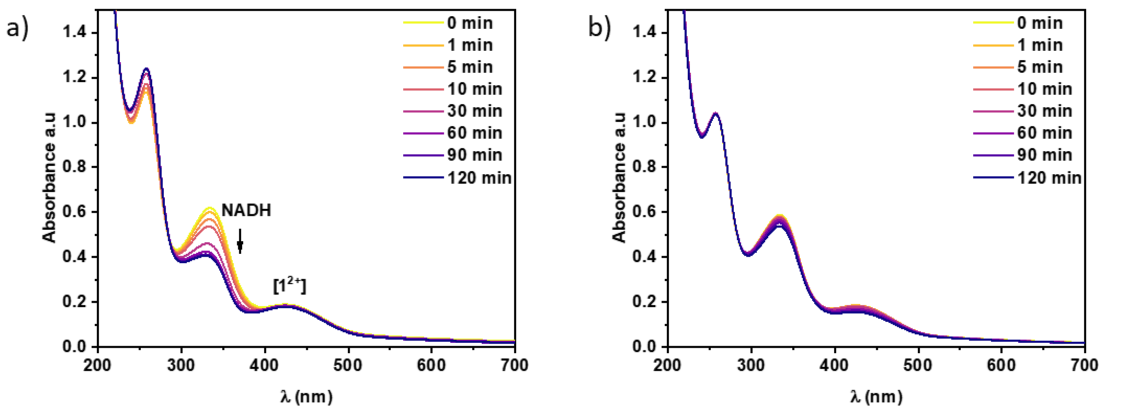

Figure S27. Temporal UV-vis spectra in DPPC:(14:0 PEG2000 PE):**[1]**$^{2+}$= 100:1:1 (NADH/**[1]**$^{2+}$/) with encapsulation of NADH (0.021 M) in liposome in phosphate buffer pH 7.0, irradiated with 470 nm LED stick a) ambient air; b) argon atmosphere.

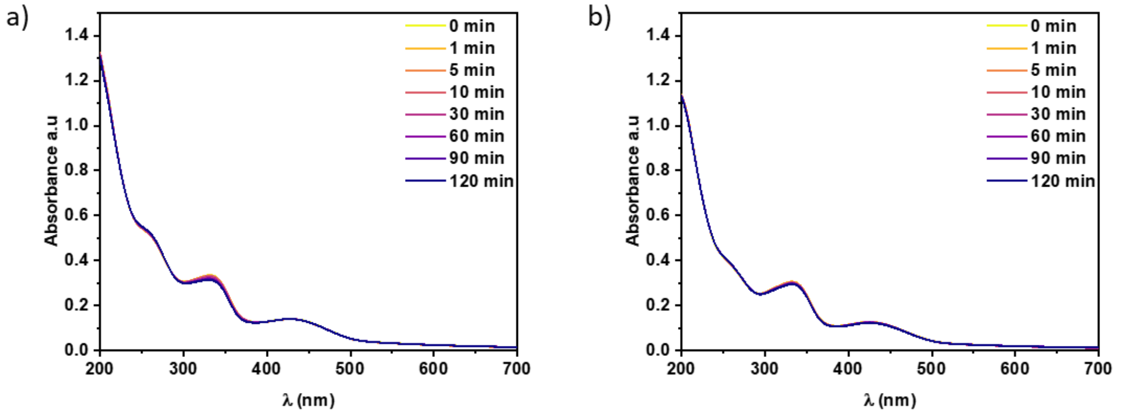

Figure S28. Temporal UV-vis spectra in DPPC:(14:0 PEG2000 PE):**[1]**$^{2+}$= 100:1:1 (/**[1]**$^{2+}$/NADH) with NADH (2 x 10^{-5} M) at the bulk solution of liposome in phosphate buffer pH 7.0, irradiated with 470 nm LED stick a) ambient air; b) argon atmosphere.

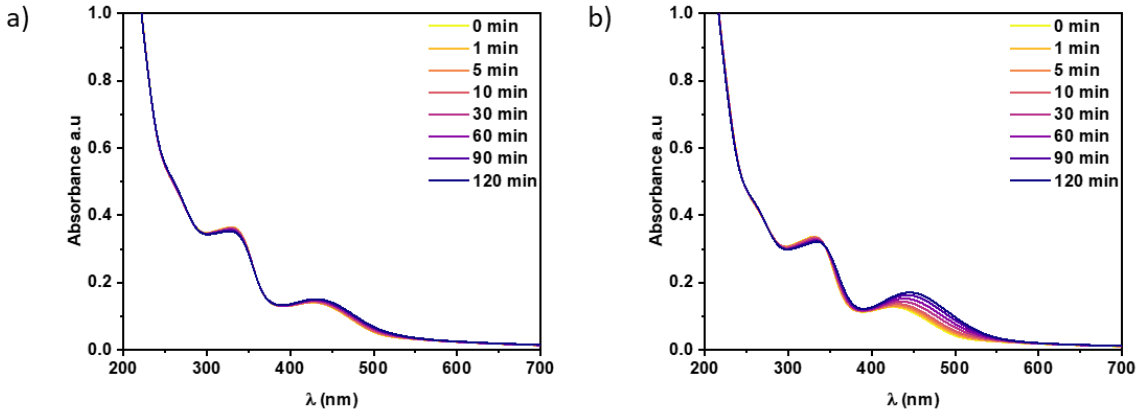

Figure S29. Temporal UV-vis spectra in DPPC:(14:0 PEG2000 PE):**[1]**$^{2+}$= 100:1:1 (/**[1]**$^{2+}$/NADH, XTT) with NADH (2 x 10^{-5} M) and XTT (2 x 10^{-5} M) (no compartmentalization) at the bulk solution of liposome in phosphate buffer pH 7.0, irradiated with 470 nm LED stick a) ambient air; b) argon atmosphere.

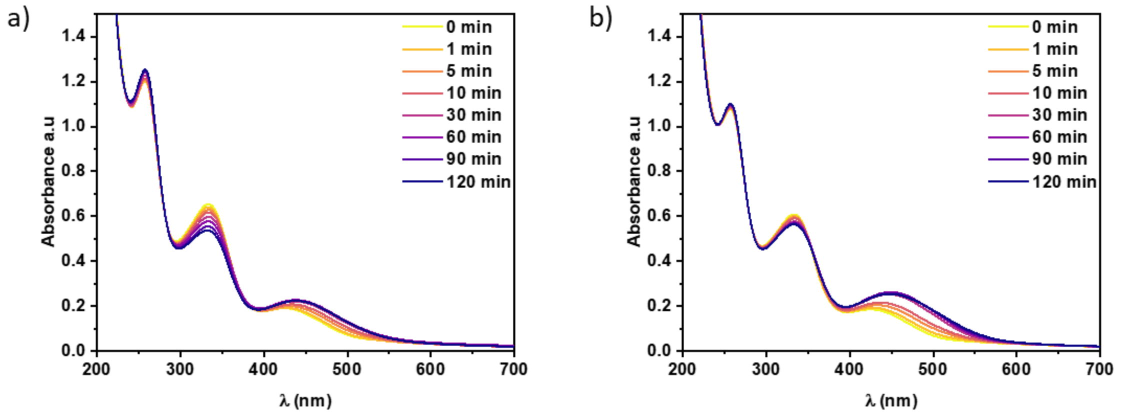

Figure S30. Temporal UV-vis spectra in DPPC:(14:0 PEG2000 PE):**[1]**$^{2+}$= 100:1:1 (NADH/**[1]**$^{2+}$/XTT) with NADH (0.021 M) encapsulated inside liposome and XTT (2 x 10^{-5} M) (no compartmentalization) at the bulk solution of liposomes in phosphate buffer pH 7.0, irradiated with 470 nm LED stick a) ambient air; b) argon atmosphere.

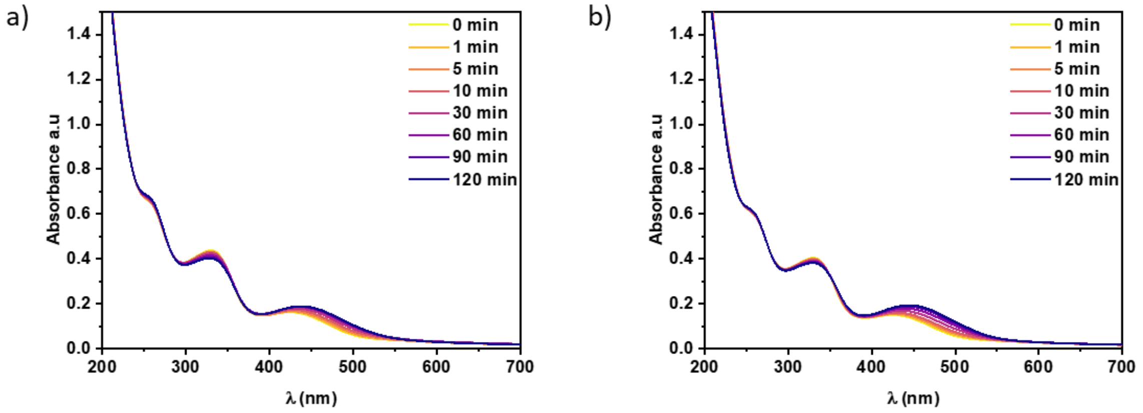

Figure S31. Temporal UV-vis spectra in DPPC:(14:0 PEG2000 PE):**[1]$^{2+}$:**18-Crown-6= 100:1:1:4. (NADH/**[1]**$^{2+}$,4.2%18-crown-6/XTT) with NADH (0.021 M) encapsulated inside liposome and XTT (2 x 10^{-5} M) (no compartmentalization) at the bulk solution of liposomes in phosphate buffer pH 7.0, irradiated with 470 nm LED stick a) ambient air; b) argon atmosphere.

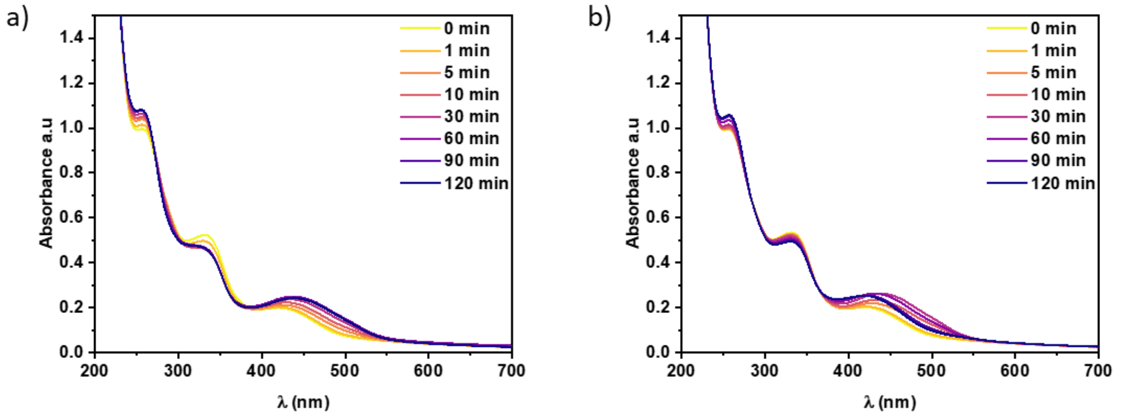

Figure S32. Temporal UV-vis spectra in DPPC:(14:0 PEG2000 PE):**[1]$^{2+}$:**Gramicidin A= 100:1:1:1 (NADH/**[1]**$^{2+}$,1%gramicidinA/XTT) with NADH (0.021 M) encapsulated inside liposome and XTT (2 x 10^{-5} M) (no compartmentalization) at the bulk solution of liposomes in phosphate buffer pH 7.0, irradiated with 470 nm LED stick a) ambient air; b) argon atmosphere.

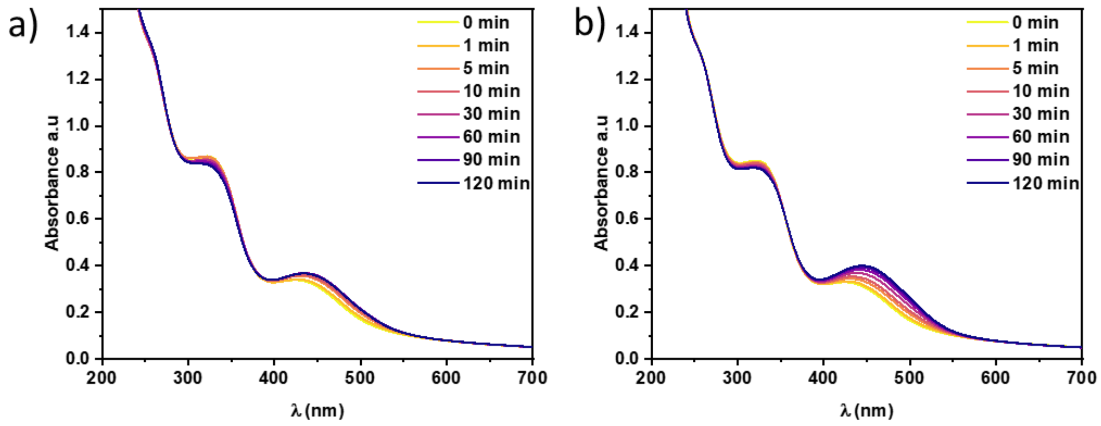

Figure S33. Temporal UV-vis spectra in DPPC:(14:0 PEG2000 PE):**[1]**$^{2+}$= 100:1:1 (XTT/**[1]**$^{2+}$/NADH)with XTT (0.009 M) encapsulated inside liposome and NADH (2 x 10^{-5} M) (no compartmentalization) at the bulk solution of liposome in phosphate buffer pH 7.0, irradiated with 470 nm LED stick a) ambient air; b) argon atmosphere.

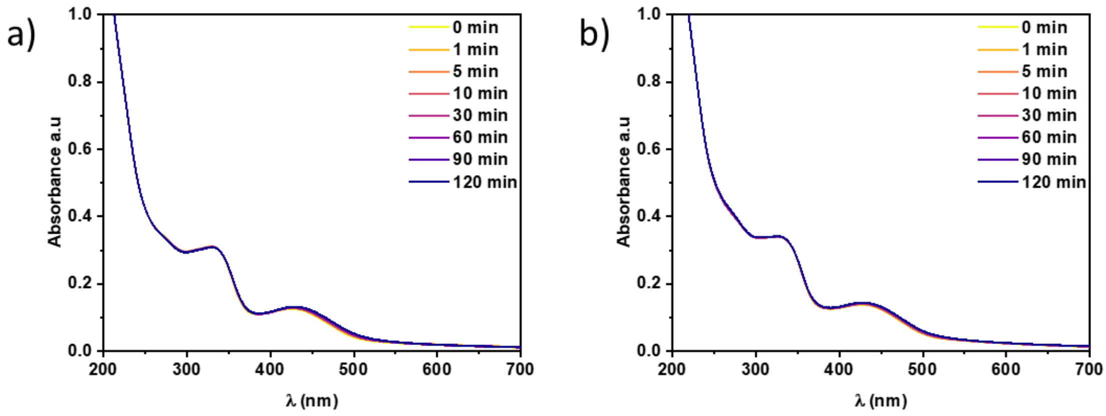

Figure S34. Temporal UV-vis spectra in DPPC:(14:0 PEG2000 PE):**[1]**$^{2+}$ = 100:1:1 (/**[1]**$^{2+}$/XTT) with XTT (2 x 10^{-5} M) (no compartmentalization) at the bulk solution of liposomes in phosphate buffer pH 7.0, irradiated with 470 nm LED stick a) ambient air; b) argon atmosphere.

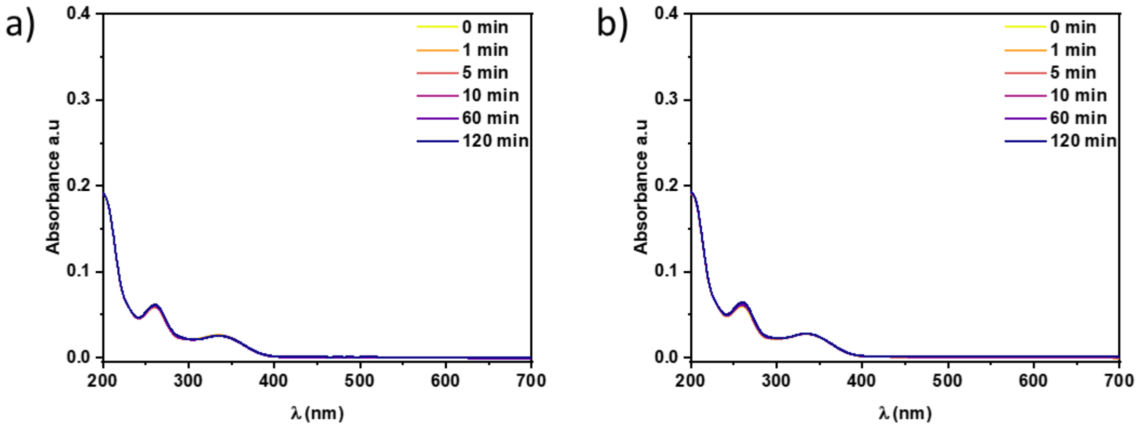

Figure S35. Temporal UV-vis spectra in DPPC:(14:0 PEG2000 PE) = 100:1 (//XTT) with XTT (2 x 10^{-5} M) (no compartmentalization, absence of **[1]**$^{2+}$) at the bulk solution of liposomes in phosphate buffer pH 7.0, irradiated with 470 nm LED stick a) ambient air; b) argon atmosphere.

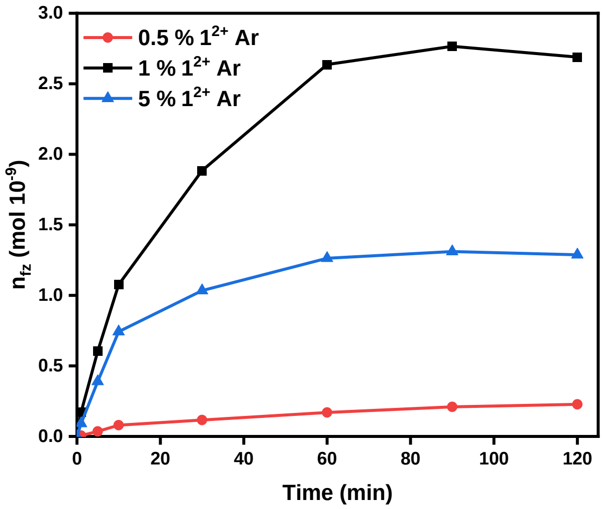

Figure S36. Formation of formazan over time with different loading of **[1]**$^{2+}$ in the DPPC with a composition of NADH/**[1]**$^{2+}$/XTT under inert atmosphere.

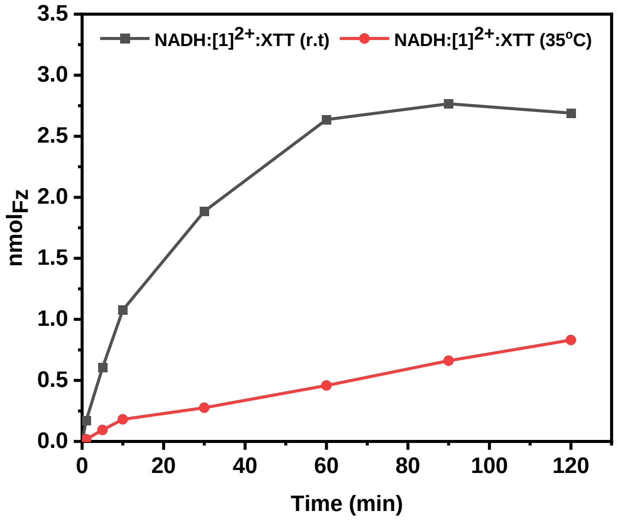

Figure S37. Formation of formazan over time at room temperature (20-25°C) and 35 °C with a composition of NADH/**[1]**$^{2+}$/XTT under an inert atmosphere Electron transfer at room temperature is more efficient. This could be related to the higher temperature, which could give the donor or acceptor electron a higher mobility, which then affects the efficiency of electron transfer, or it could be due to increased permeability around the transitions temperature (40 °C).

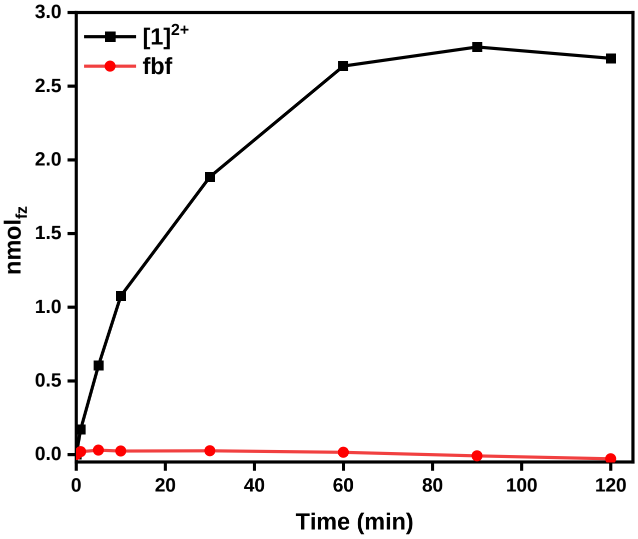

Figure S38. Formation of formazan over time with a composition of NADH/**[1]**$^{2+}$/XTT and NADH/**fbf**/XTT under an inert atmosphere.

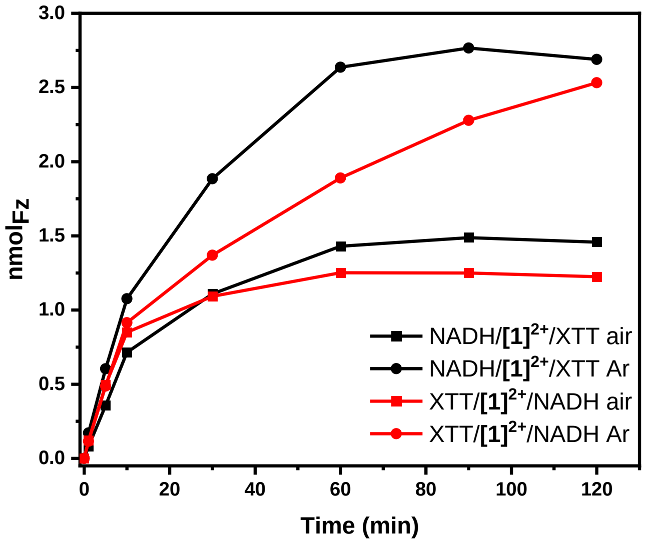

Figure S39. Formation of formazan over time with a composition of NADH/**[1]**$^{2+}$/XTT and XTT/**[1]**$^{2+}$/NADH under an inert and ambient air atmosphere.

S12. Nanosecond transient absorption spectra

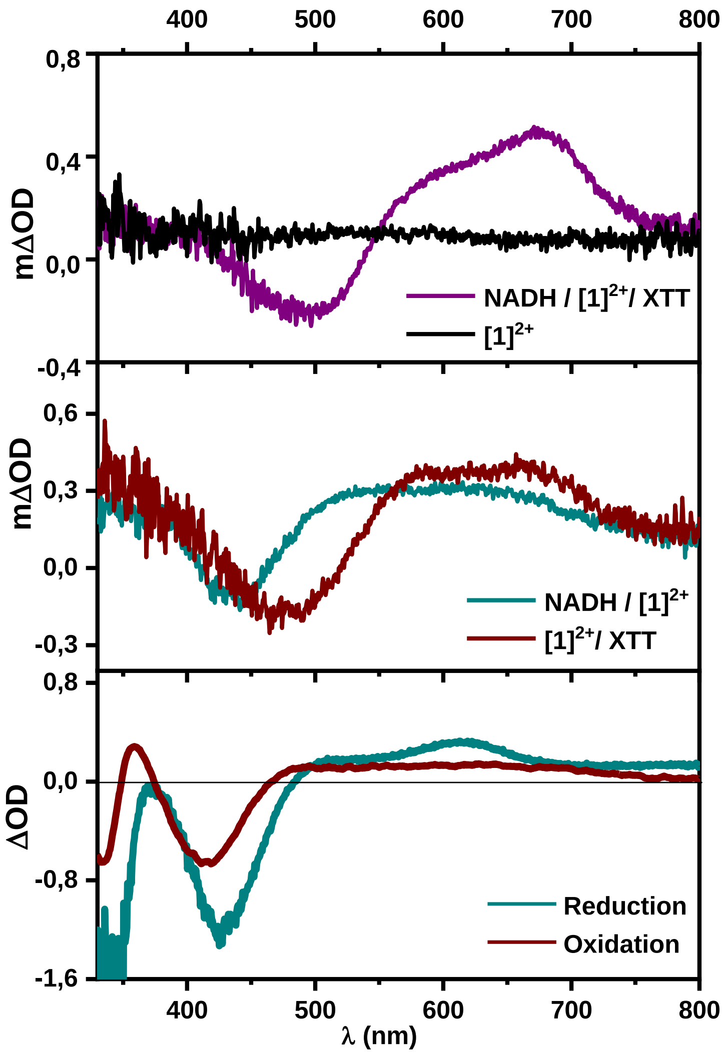

Figure S40. Transient absorption spectrum of 1% **[1]**$^{2+}$ in DPPC (black), spectrum of 1% **[1]**$^{2+}$ in DPPC with encapsulated NADH and XTT in bulk (purple), encapsulated NADH inside DPPC liposome with 1% of **[1]**$^{2+}$ (red), and DPPC liposome with 1% of **[1]**$^{2+}$ XTT at the bulk (blue) after excitation at 420 nm laser pulse of ~10 ns duration after a delay of 1 μs. All measurements were performed in argon atmosphere at 20 °C. (bottom) Spectro-electrochemical UV–vis, the spectrum of **[1]**$^{2+}$ in dry acetonitrile for reduction (red) at an applied potential of -1.8 V vs Fc/Fc^{+} in dry DMF for oxidation (blue) at an applied potential of 2.0 V vs Fc/Fc^{+}, the electrolyte containing 0.1 M tetra-n-butylammonium hexafluorophosphate ($TBAPF_6$).

[1]$^{2+}$ at DPPC

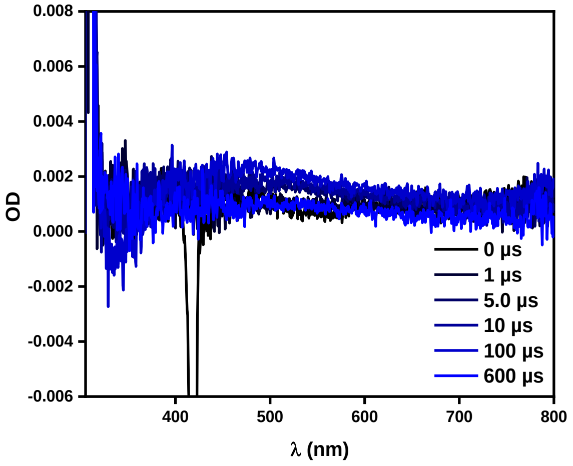

Figure S41. Temporal nanosecond transient absorption spectra of DPPC:(14:0 PEG2000 PE):**[1]**$^{2+}$= 100:1:1 liposomes in phosphate buffer pH 7.0 upon 420 nm pump excitation.

Encapsulated NADH in the liposome

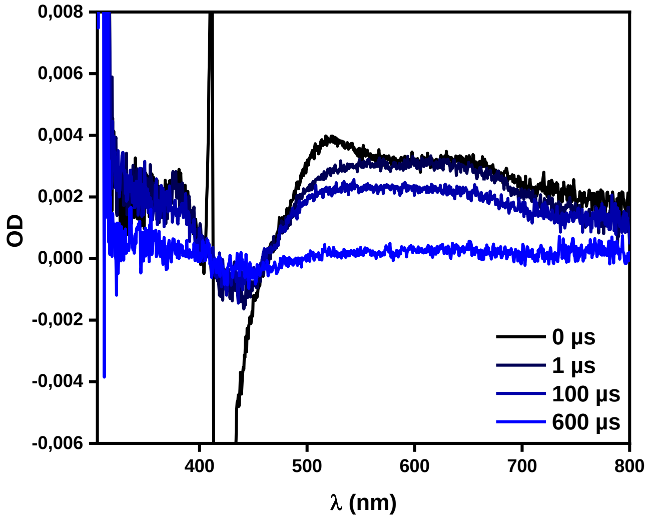

Figure S42. Temporal nanosecond transient absorption spectrum of encapsulated NADH (theoretical value of 22.5 mM) DPPC:(14:0 PEG2000 PE):**[1]**$^{2+}$= 100:1:1 liposomes in phosphate buffer pH 7.0 upon 420 nm pump excitation.

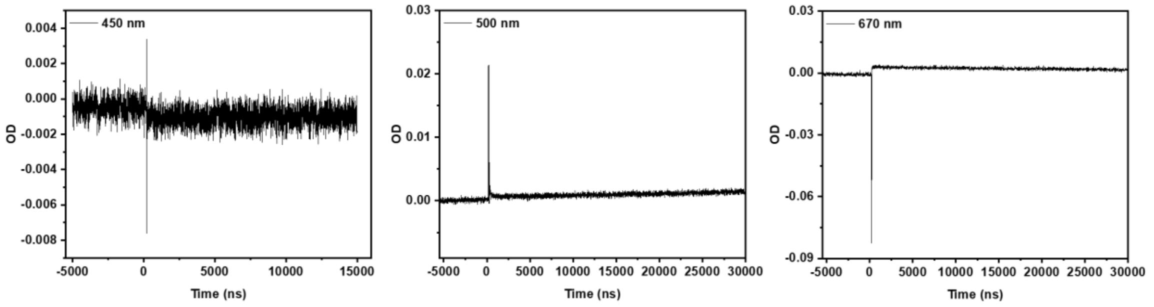

Figure S43. Time traces of the excited states of encapsulated NADH (theoretical value of 22.5 mM) DPPC:(14:0 PEG2000 PE):**[1]**$^{2+}$= 100:1:1 liposomes in phosphate buffer pH 7.0 at 450nm, 500 nm and 670 nm.

XTT at the bulk solution of liposome

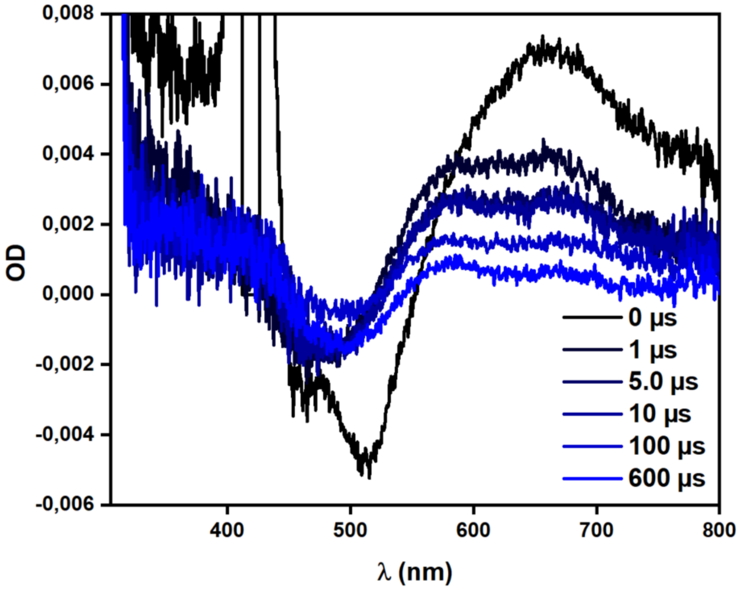

Figure S44. Temporal nanosecond transient absorption spectrum of DPPC:(14:0 PEG2000 PE):**[1]**$^{2+}$= 100:1:1 liposomes in phosphate buffer pH 7.0 with XTT (80 µM) at the bulk solution of liposome upon 420 nm pump excitation.

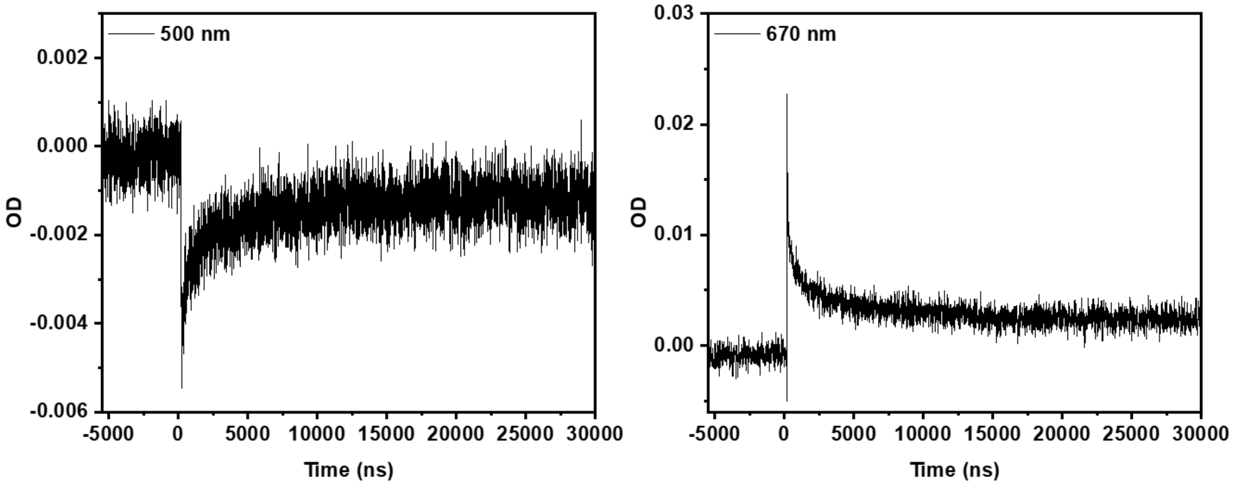

Figure S45. Time traces of the excited states of DPPC:(14:0 PEG2000 PE):**[1]**$^{2+}$= 100:1:1 liposomes in phosphate buffer pH 7.0 with XTT (80 μM) at the bulk solution of liposome at 500 nm and 670 nm.

Encapsulated NADH in the liposome and XTT at the bulk solution

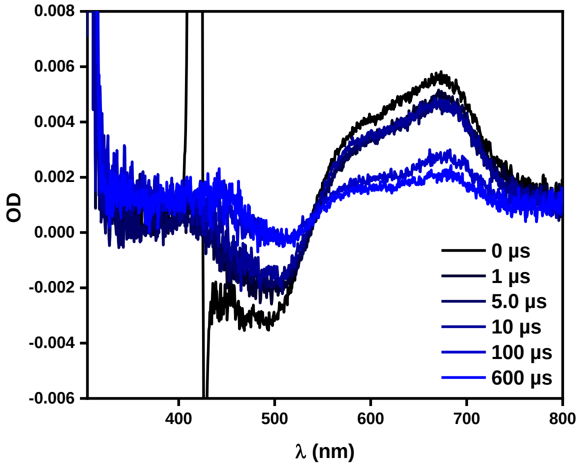

Figure S46.Temporal nanosecond transient absorption spectrum of encapsulated NADH (theoretical value of 22.5 mM) DPPC:(14:0 PEG2000 PE):**[1]**$^{2+}$= 100:1:1 liposomes in phosphate buffer pH 7.0 with XTT (80 μM) at the bulk solution of liposome upon 420 nm pump excitation.

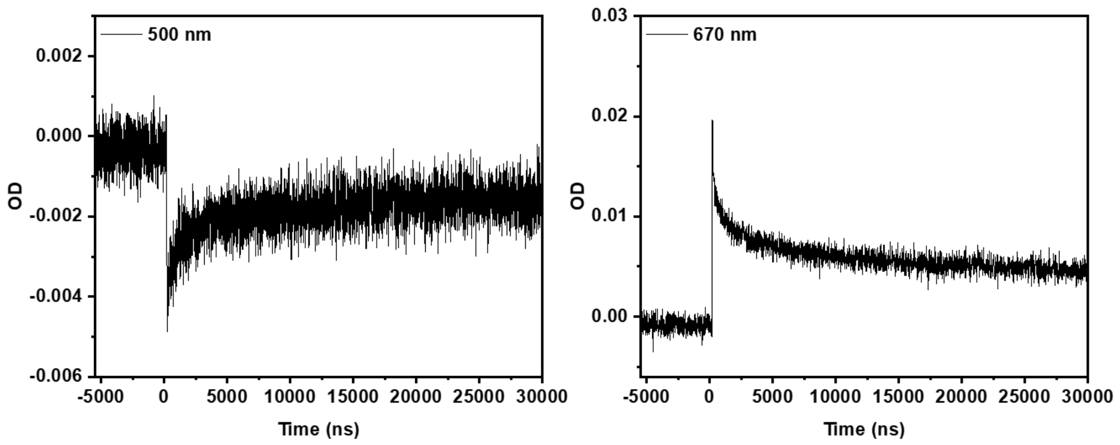

Figure S47. Time traces of the excited states of encapsulated NADH (theoretical value of 22.5 mM) DPPC:(14:0 PEG2000 PE):**[1]**$^{2+}$= 100:1:1 liposomes in phosphate buffer pH 7.0 with XTT (80 μM) at the bulk solution of liposome at 500 nm and 670 nm.

S13. Stern-Volmer quenching experiment

Liposomes were prepared as described in the methods section.

In cuvette:

V = 0.5 mL

[1]$^{2+}$= 10 μM

[DPPC] = 100 μM

[14:0 PEG2000 PE] = 1 μM

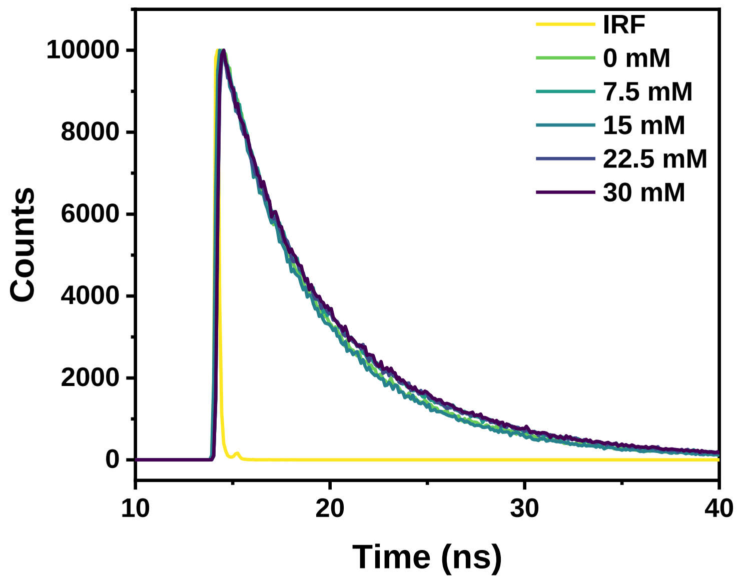

Figure S48. Kinetic traces of luminescence decay upon excitation at 450 nm with various concentrations of encapsulated NADH inside liposome as a quencher.

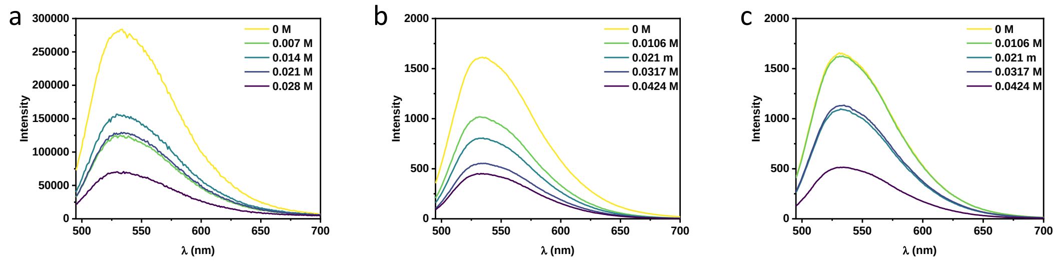

Figure S49. Luminescence quenching upon encapsulation of various concentrations of NADH inside liposome as a quencher (triplicates a, b, and c).

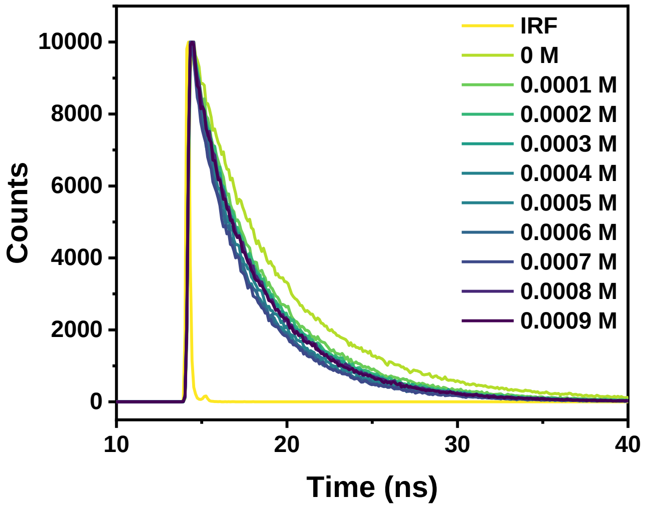

Figure S50. Kinetic traces of luminescence decay upon excitation at 450 nm with various concentrations of XTT as a quencher.

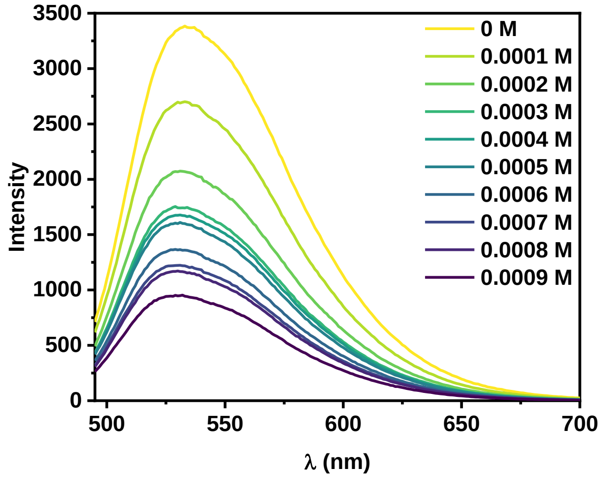

Figure S51. Luminescence quenching upon the addition of various concentrations of XTT as a quencher.

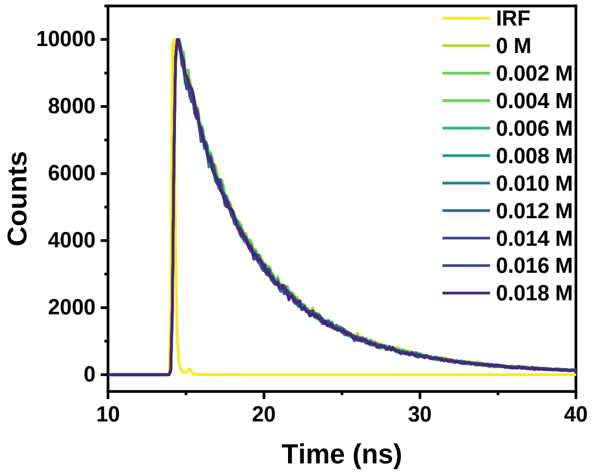

Figure S52. Kinetic traces of luminescence decay upon excitation at 450 nm with various concentrations of NADH as a quencher at the bulk.

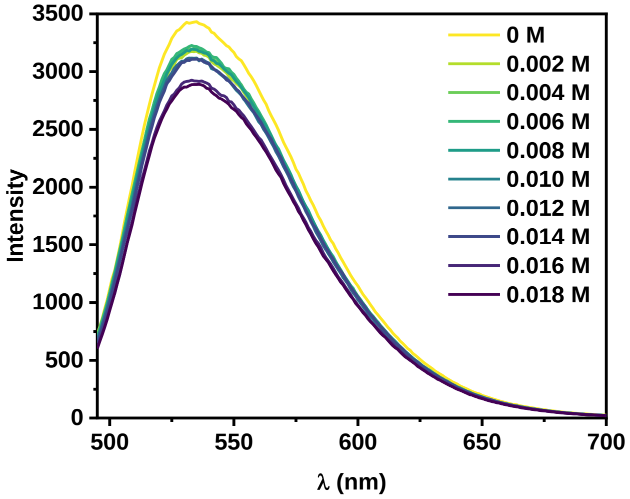

Figure S53. Luminescence quenching upon the addition of various concentrations of NADH as a quencher at the bulk.

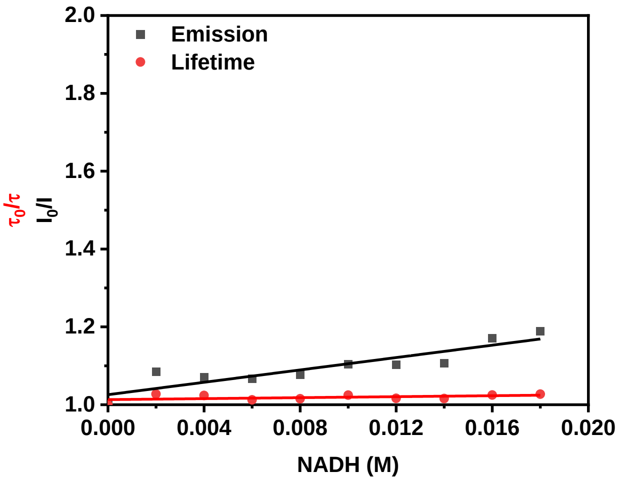

Figure. S54. SV-Plot from the quenching experiment with NADH located at the bulk solution.

S14. Proposed mechanism in the presence of oxygen, photooxidation or photoreduction

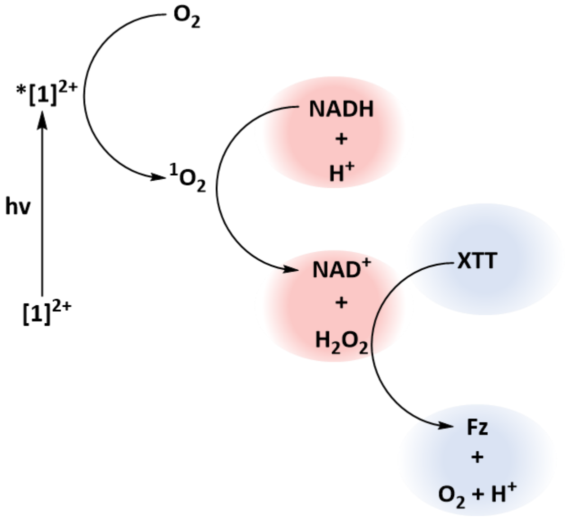

Figure. S55. Proposed mechanism in the presence of oxygen.

S15. Coordinates of $[1]^{2+}$

C	-0.47536	-1.15533	0.26542
C	-1.38655	-0.27254	-0.24406
C	0.91016	-0.86985	0.35754
C	1.45861	0.31269	-0.05600
C	0.54624	1.25715	-0.61697
C	-0.85606	0.96896	-0.70864
C	2.90614	0.58500	0.06655
N	0.86088	2.44842	-1.14024
H	-0.82224	-2.10816	0.64529
H	1.55187	-1.61885	0.80445
N	-1.54591	1.95383	-1.29695
C	-2.82849	-0.58881	-0.31490
S	-0.49723	3.09734	-1.67121
C	3.82969	-0.41352	-0.25370
C	5.17927	-0.17180	-0.10042
C	5.62786	1.06280	0.37112
C	4.72313	2.06427	0.69034
C	3.36877	1.81918	0.53271
H	3.48013	-1.36624	-0.63470
C	6.34203	-1.10380	-0.39003
H	5.06059	3.02444	1.06071
H	2.65515	2.59162	0.78459
C	7.51939	-0.22606	-0.00563
C	8.86388	-0.53094	-0.04787
C	9.80260	0.42682	0.34404
C	9.36077	1.68007	0.77522
C	8.01065	1.98917	0.82275
C	7.09050	1.02970	0.42947
H	9.19538	-1.50038	-0.40214
C	11.24964	0.11585	0.30721
H	10.08444	2.41742	1.09929
H	7.68893	2.96341	1.16930
C	6.39364	-1.48735	-1.87406
C	6.27779	-2.36415	0.48129
H	6.24150	-2.10475	1.54040
H	7.15444	-2.99125	0.30885
H	5.38895	-2.94923	0.23823
H	6.43941	-0.59877	-2.50544
H	5.50697	-2.06140	-2.14907
H	7.27229	-2.10206	-2.07796
C	12.17995	1.06466	-0.10171
C	13.53684	0.78278	-0.13280
C	13.98012	-0.46946	0.24878
C	13.07196	-1.43516	0.65732
C	11.72345	-1.14046	0.68465
H	11.84933	2.04386	-0.42178
H	14.21089	1.55616	-0.46398
N	15.43169	-0.81954	0.23720
H	13.39044	-2.42015	0.96777
H	11.03217	-1.89950	1.02603
C	16.29536	0.31212	-0.21764
C	15.86939	-1.19503	1.62268
H	17.32519	-0.03089	-0.19140
H	16.17351	1.15413	0.45676
H	16.02733	0.58591	-1.23326
H	15.67434	-0.35423	2.28212

H	16.93200	-1.41998	1.59523
H	15.31676	-2.06741	1.95341
C	15.66368	-1.97220	-0.69530
H	15.32844	-1.68155	-1.68683
H	15.10631	-2.83575	-0.34931
H	16.72667	-2.19738	-0.70239
C	-3.24484	-1.84762	-0.75634
C	-3.78694	0.34823	0.07986
C	-5.12389	0.01457	0.03367
C	-5.52782	-1.24649	-0.41002
C	-4.58848	-2.18484	-0.80803
H	-3.47473	1.32432	0.42889
C	-6.31813	0.86970	0.41632
C	-6.98998	-1.30714	-0.35498
H	-2.50230	-2.56648	-1.07960
H	-4.88962	-3.16346	-1.16090
C	-7.46239	-0.08499	0.12826
C	-8.81583	0.13162	0.28137
C	-9.72002	-0.87849	-0.05587
C	-9.23543	-2.09571	-0.54066
C	-7.87578	-2.31956	-0.69049
H	-9.18152	1.08707	0.63977
C	-11.17590	-0.65852	0.09843
H	-9.93285	-2.88727	-0.78488
H	-7.52041	-3.27374	-1.05934
C	-11.68604	0.02265	1.19786
C	-13.04677	0.24092	1.34762
C	-13.92018	-0.22800	0.38446
C	-13.43659	-0.91225	-0.72107
C	-12.07898	-1.12301	-0.85739
H	-11.71834	-1.64141	-1.73587
H	-11.01764	0.38079	1.96967
H	-13.38578	0.77000	2.22373
N	-15.39273	-0.00673	0.49340
H	-14.09598	-1.28173	-1.49355
C	-15.77825	0.70242	1.75090
C	-16.10768	-1.32571	0.48036
H	-15.31401	1.68332	1.77008
H	-15.47169	0.10975	2.60738
H	-16.85849	0.81091	1.74755
H	-15.74193	-1.92125	1.31180
H	-15.91606	-1.83318	-0.45841
H	-17.17237	-1.13640	0.58573
C	-15.85789	0.82901	-0.66280
H	-15.32212	1.77370	-0.63816
H	-16.92685	0.99282	-0.55710
H	-15.65395	0.30595	-1.59082
C	-6.40501	2.12552	-0.45988
C	-6.27586	1.25950	1.89882
H	-6.21394	0.37423	2.53349
H	-7.17325	1.81808	2.17119
H	-5.40885	1.89096	2.10109
H	-6.43806	1.86130	-1.51791
H	-5.53779	2.76658	-0.29127
H	-7.30257	2.69767	-0.21802

References

(1) M. J.Frisch, G. W.Trucks, H. B.Schlegel, G. E.Scuseria, M. A.Robb, J. R.Cheeseman, G.Scalmani, V.Barone, G. A.Petersson, H.Nakatsuji, X.Li, M.Caricato, A. V.Marenich, J.Bloino, B. G.Janesko, R.Gomperts, B.Mennucci, H. P.Hratchian, J. V.Ortiz, A. F.Izmaylov, J. L.Sonnenberg, F. DingWilliams, F.Lipparini, F.Egidi, J.Goings, B.Peng, A.Petrone, T.Henderson, D.Ranasinghe, V. G.Zakrzewski, J.Gao, N.Rega, G.Zheng, W.Liang, M.Hada, M.Ehara, K.Toyota, R.Fukuda, J.Hasegawa, M.Ishida, T.Nakajima, Y.Honda, O.Kitao, H.Nakai, T.Vreven, K.Throssell, J. A.Montgomery Jr., J. E.Peralta, F.Ogliaro, M. J.Bearpark, J. J.Heyd, E. N.Brothers, K. N.Kudin, V. N.Staroverov, T. A.Keith, R.Kobayashi, J.Normand, K.Raghavachari, A. P.Rendell, J. C.Burant, S. S.Iyengar, J.Tomasi, M.Cossi, J. M.Millam, M.Klene, C.Adamo, R.Cammi, J. W.Ochterski, R. L.Martin, K.Morokuma, O.Farkas, J. B.Foresman and D. J.Fox, Gaussian 16, Revision C.01, Gaussian, Inc., Wallingford, CT, 2016. *Citation | Gaussian.com*. https://gaussian.com/citation/ (accessed 2023-12-12).

(2) Perdew, J. P. Density-Functional Approximation for the Correlation Energy of the Inhomogeneous Electron Gas. *Phys. Rev. B* **1986**, *33* (12), 8822–8824. https://doi.org/10.1103/PhysRevB.33.8822.

(3) Lee, C.; Yang, W.; Parr, R. G. Development of the Colle-Salvetti Correlation-Energy Formula into a Functional of the Electron Density. *Phys. Rev. B* **1988**, *37* (2), 785–789. https://doi.org/10.1103/PhysRevB.37.785.

(4) Becke, A. D. Density-functional Thermochemistry. III. The Role of Exact Exchange. *The Journal of Chemical Physics* **1993**, *98* (7), 5648–5652. https://doi.org/10.1063/1.464913.

(5) Adamo, C.; Barone, V. Toward Reliable Density Functional Methods without Adjustable Parameters: The PBE0 Model. *The Journal of Chemical Physics* **1999**, *110* (13), 6158–6170. https://doi.org/10.1063/1.478522.

(6) Perdew, J. P.; Burke, K.; Ernzerhof, M. Generalized Gradient Approximation Made Simple [Phys. Rev. Lett. 77, 3865 (1996)]. *Phys. Rev. Lett.* **1997**, *78* (7), 1396–1396. https://doi.org/10.1103/PhysRevLett.78.1396.

(7) Perdew, J. P.; Burke, K.; Ernzerhof, M. Generalized Gradient Approximation Made Simple. *Phys. Rev. Lett.* **1996**, *77* (18), 3865–3868. https://doi.org/10.1103/PhysRevLett.77.3865.

(8) Yanai, T.; Tew, D. P.; Handy, N. C. A New Hybrid Exchange–Correlation Functional Using the Coulomb-Attenuating Method (CAM-B3LYP). *Chemical Physics Letters* **2004**, *393* (1), 51–57. https://doi.org/10.1016/j.cplett.2004.06.011.

(9) Vydrov, O. A.; Scuseria, G. E. Assessment of a Long-Range Corrected Hybrid Functional. *The Journal of Chemical Physics* **2006**, *125* (23), 234109. https://doi.org/10.1063/1.2409292.

(10) Vreven, T.; Frisch, M. J.; Kudin, K. N.; Schlegel, H. B.; Morokuma, K. Geometry Optimization with QM/MM Methods II: Explicit Quadratic Coupling. *Molecular Physics* **2006**, *104* (5–7), 701–714. https://doi.org/10.1080/00268970500417846.

(11) Vydrov, O. A.; Scuseria, G. E.; Perdew, J. P. Tests of Functionals for Systems with Fractional Electron Number. *The Journal of Chemical Physics* **2007**, *126* (15), 154109. https://doi.org/10.1063/1.2723119.

(12) *Can short-range hybrids describe long-range-dependent properties? | The Journal of Chemical Physics | AIP Publishing*. https://pubs.aip.org/aip/jcp/article/131/4/044108/838663/Can-short-range-hybrids-describe-long-range (accessed 2024-05-14).

(13) Chai, J.-D.; Head-Gordon, M. Long-Range Corrected Hybrid Density Functionals with Damped Atom–Atom Dispersion Corrections. *Phys. Chem. Chem. Phys.* **2008**, *10* (44), 6615–6620. https://doi.org/10.1039/B810189B.

(14) Grimme, S.; Ehrlich, S.; Goerigk, L. Effect of the Damping Function in Dispersion Corrected Density Functional Theory. *Journal of Computational Chemistry* **2011**, *32* (7), 1456–1465. https://doi.org/10.1002/jcc.21759.

(15) Weigend, F. Accurate Coulomb-Fitting Basis Sets for H to Rn. *Phys. Chem. Chem. Phys.* **2006**, *8* (9), 1057–1065. https://doi.org/10.1039/B515623H.

(16) Weigend, F.; Ahlrichs, R. Balanced Basis Sets of Split Valence, Triple Zeta Valence and Quadruple Zeta Valence Quality for H to Rn: Design and Assessment of Accuracy. *Phys. Chem. Chem. Phys.* **2005**, *7* (18), 3297–3305. https://doi.org/10.1039/B508541A.
(17) Cossi, M.; Rega, N.; Scalmani, G.; Barone, V. Energies, Structures, and Electronic Properties of Molecules in Solution with the C-PCM Solvation Model. *Journal of Computational Chemistry* **2003**, *24* (6), 669–681. https://doi.org/10.1002/jcc.10189.
(18) Barone, V.; Cossi, M. Quantum Calculation of Molecular Energies and Energy Gradients in Solution by a Conductor Solvent Model. *J. Phys. Chem. A* **1998**, *102* (11), 1995–2001. https://doi.org/10.1021/jp9716997.
(19) Jacquemin, D.; Mennucci, B.; Adamo, C. Excited-State Calculations with TD-DFT: From Benchmarks to Simulations in Complex Environments. *Phys. Chem. Chem. Phys.* **2011**, *13* (38), 16987–16998. https://doi.org/10.1039/C1CP22144B.
(20) Jacquemin, D.; Wathelet, V.; Perpète, E. A.; Adamo, C. Extensive TD-DFT Benchmark: Singlet-Excited States of Organic Molecules. *J. Chem. Theory Comput.* **2009**, *5* (9), 2420–2435. https://doi.org/10.1021/ct900298e.
(21) Mai, S.; Avagliano, D.; Heindl, M.; Marquetand, P.; Menger, M. F. S. J.; Oppel, M.; Plasser, F.; Polonius, S.; Ruckenbauer, M.; Shu, Y.; Truhlar, D. G.; Zhang, L.; Zobel, P.; González, L. SHARC3.0: Surface Hopping Including Arbitrary Couplings – Program Package for Non-Adiabatic Dynamics, 2023. https://doi.org/10.5281/zenodo.7828641.
(22) Koopmans, T. Über Die Zuordnung von Wellenfunktionen Und Eigenwerten Zu Den Einzelnen Elektronen Eines Atoms. *Physica* **1934**, *1* (1), 104–113. https://doi.org/10.1016/S0031-8914(34)90011-2.
(23) Kasha, M. Characterization of Electronic Transitions in Complex Molecules. *Discuss. Faraday Soc.* **1950**, *9* (0), 14–19. https://doi.org/10.1039/DF9500900014.
(24) Alcoba, D. R.; Lain, L.; Torre, A.; Oña, O. B.; Tiznado, W. Ground and Excited State Similarity Studies by Means of Fukui and Dual-Descriptor Matrices. *Chemical Physics Letters* **2012**, *549*, 103–107. https://doi.org/10.1016/j.cplett.2012.08.030.
(25) *Jmol: an open-source Java viewer for chemical structures in 3D*. https://jmol.sourceforge.net/ (accessed 2023-12-12).
(26) Plasser, F. TheoDORE: A Toolbox for a Detailed and Automated Analysis of Electronic Excited State Computations. *The Journal of Chemical Physics* **2020**, *152* (8), 084108. https://doi.org/10.1063/1.5143076.
(27) Sinambela, N.; Jacobi, R.; Hernández-Castillo, D.; Hofmeister, E.; Hagmeyer, N.; Dietzek, B.; González, L.; Pannwitz, A. Alignment and Photooxidation Dynamics of a Perylene Diimide Chromophore in Lipid Bilayers. *Mol. Syst. Des. Eng.* **2023**, 10.1039.D2ME00243D. https://doi.org/10.1039/D2ME00243D.
(28) Jo, S.; Kim, T.; Iyer, V. G.; Im, W. CHARMM-GUI: A Web-Based Graphical User Interface for CHARMM. *Journal of Computational Chemistry* **2008**, *29* (11), 1859–1865. https://doi.org/10.1002/jcc.20945.
(29) D.A. Case, H.M. Aktulga, K. Belfon, I.Y. Ben-Shalom, J.T. Berryman, S.R. Brozell, D.S. Cerutti, T.E. Cheatham, III, G.A. Cisneros, V.W.D. Cruzeiro, T.A. Darden, N. Forouzesh, G. Giambaşu, T. Giese, M.K. Gilson, H. Gohlke, A.W. Goetz, J. Harris, S. Izadi, S.A. Izmailov, K. Kasavajhala, M.C. Kaymak, E. King, A. Kovalenko, T. Kurtzman, T.S. Lee, P. Li, C. Lin, J. Liu, T. Luchko, R. Luo, M. Machado, V. Man, M. Manathunga, K.M. Merz, Y. Miao, O. Mikhailovskii, G. Monard, H. Nguyen, K.A. O'Hearn, A. Onufriev, F. Pan, S. Pantano, R. Qi, A. Rahnamoun, D.R. Roe, A. Roitberg, C. Sagui, S. Schott-Verdugo, A. Shajan, J. Shen, C.L. Simmerling, N.R. Skrynnikov, J. Smith, J. Swails, R.C. Walker, J. Wang, J. Wang, H. Wei, X. Wu, Y. Wu, Y. Xiong, Y. Xue, D.M. York, S. Zhao, Q. Zhu, and P.A. Kollman (2022), Amber 2022, University of California, San Francisco. Amber.

(30) Dickson, C. J.; Walker, R. C.; Gould, I. R. Lipid21: Complex Lipid Membrane Simulations with AMBER. *J. Chem. Theory Comput.* **2022**, *18* (3), 1726–1736. https://doi.org/10.1021/acs.jctc.1c01217.
(31) Izadi, S.; Anandakrishnan, R.; Onufriev, A. V. Building Water Models: A Different Approach. *J. Phys. Chem. Lett.* **2014**, *5* (21), 3863–3871. https://doi.org/10.1021/jz501780a.
(32) Ryckaert, J.-P.; Ciccotti, G.; Berendsen, H. J. C. Numerical Integration of the Cartesian Equations of Motion of a System with Constraints: Molecular Dynamics of n-Alkanes. *Journal of Computational Physics* **1977**, *23* (3), 327–341. https://doi.org/10.1016/0021-9991(77)90098-5.
(33) Berendsen, H. J. C.; Postma, J. P. M.; van Gunsteren, W. F.; DiNola, A.; Haak, J. R. Molecular Dynamics with Coupling to an External Bath. *The Journal of Chemical Physics* **1984**, *81* (8), 3684–3690. https://doi.org/10.1063/1.448118.
(34) Götz, A. W.; Williamson, M. J.; Xu, D.; Poole, D.; Le Grand, S.; Walker, R. C. Routine Microsecond Molecular Dynamics Simulations with AMBER on GPUs. 1. Generalized Born. *J. Chem. Theory Comput.* **2012**, *8* (5), 1542–1555. https://doi.org/10.1021/ct200909j.
(35) Salomon-Ferrer, R.; Götz, A. W.; Poole, D.; Le Grand, S.; Walker, R. C. Routine Microsecond Molecular Dynamics Simulations with AMBER on GPUs. 2. Explicit Solvent Particle Mesh Ewald. *J. Chem. Theory Comput.* **2013**, *9* (9), 3878–3888. https://doi.org/10.1021/ct400314y.
(36) Le Grand, S.; Götz, A. W.; Walker, R. C. SPFP: Speed without Compromise—A Mixed Precision Model for GPU Accelerated Molecular Dynamics Simulations. *Computer Physics Communications* **2013**, *184* (2), 374–380. https://doi.org/10.1016/j.cpc.2012.09.022.
(37) Humphrey, W.; Dalke, A.; Schulten, K. VMD: Visual Molecular Dynamics. *Journal of Molecular Graphics* **1996**, *14* (1), 33–38. https://doi.org/10.1016/0263-7855(96)00018-5.
(38) Dutta, S.; Watson, B.; Mattoo, S.; Rochet, J.-C. Calcein Release Assay to Measure Membrane Permeabilization by Recombinant Alpha-Synuclein. *BIO-PROTOCOL* **2020**, *10* (14). https://doi.org/10.21769/BioProtoc.3690.
(39) Maherani, B.; Arab-Tehrany, E.; Kheirolomoom, A.; Geny, D.; Linder, M. Calcein Release Behavior from Liposomal Bilayer; Influence of Physicochemical/Mechanical/Structural Properties of Lipids. *Biochimie* **2013**, *95* (11), 2018–2033. https://doi.org/10.1016/j.biochi.2013.07.006.
(40) Pavlishchuk, V. V.; Addison, A. W. Conversion Constants for Redox Potentials Measured versus Different Reference Electrodes in Acetonitrile Solutions at 25°C. *Inorganica Chimica Acta* **2000**, *298* (1), 97–102. https://doi.org/10.1016/S0020-1693(99)00407-7.
(41) Ishiki, K.; Nguyen, D. Q.; Morishita, A.; Shiigi, H.; Nagaoka, T. Electrochemical Detection of Viable Bacterial Cells Using a Tetrazolium Salt. *Anal. Chem.* **2018**, *90* (18), 10903–10909. https://doi.org/10.1021/acs.analchem.8b02404.
(42) Smith, T. J.; Stevenson, K. J. 4 - Reference Electrodes. In *Handbook of Electrochemistry*; Zoski, C. G., Ed.; Elsevier: Amsterdam, 2007; pp 73–110. https://doi.org/10.1016/B978-044451958-0.50005-7.
(43) Lin, K.-C.; Chen, S.-M. Reversible Cyclic Voltammetry of the NADH/NAD+ Redox System on Hybrid Poly(Luminol)/FAD Film Modified Electrodes. *Journal of Electroanalytical Chemistry* **2006**, *589* (1), 52–59. https://doi.org/10.1016/j.jelechem.2006.01.010.
(44) Tschierlei, S.; Karnahl, M.; Presselt, M.; Dietzek, B.; Guthmuller, J.; González, L.; Schmitt, M.; Rau, S.; Popp, J. Photochemisches Schicksal: Der Erste Schritt Bestimmt Die Effizienz Der H2-Bildung Mit Einem Supramolekularen Photokatalysator. *Angewandte Chemie* **2010**, *122* (23), 4073–4076. https://doi.org/10.1002/ange.200906595.
(45) Sutherland, M. W.; Learmonth, B. A. The Tetrazolium Dyes MTS and XTT Provide New Quantitative Assays for Superoxide and Superoxide Dismutase. *Free Radical Research* **1997**, *27* (3), 283–289. https://doi.org/10.3109/10715769709065766.
